# The *Drosophila* tumour suppressor Lgl and Vap33 activate the Hippo pathway through a dual mechanism

**DOI:** 10.1242/jcs.261917

**Published:** 2024-02-16

**Authors:** Marta Portela, Swastik Mukherjee, Sayantanee Paul, John E. La Marca, Linda M. Parsons, Alexey Veraksa, Helena E. Richardson

**Affiliations:** ^1^ Department of Biochemistry & Chemistry, La Trobe Institute for Molecular Science, La Trobe University, Melbourne, Victoria, 3086, Australia; ^2^ Cell Cycle and Development Laboratory, Peter MacCallum Cancer Centre, Melbourne, Victoria, 3002, Australia; ^3^ Department of Biology, University of Massachusetts Boston, Boston, MA 02125, USA; ^4^ Blood Cells and Blood Cancer Division, Water and Eliza Hall Institute, Melbourne, Victoria, 3052, Australia; ^5^ Department of Medical Biology, University of Melbourne, Melbourne, Victoria, 3010, Australia; ^6^ Genome Engineering and Cancer Modelling Program, Olivia Newton-John Cancer Research Institute, Melbourne, Victoria, 3084, Australia; ^7^ Sir Peter MacCallum Department of Oncology, Department of Anatomy and Neuroscience, Department of Biochemistry and Molecular Biology, University of Melbourne, Melbourne, Victoria, 3010, Australia

**Keywords:** *Drosophila*, *lgl*, Vap33, Hippo pathway, RtGEF, Git, Arf79F, V-ATPase

## Abstract

The tumour suppressor, Lethal (2) giant larvae [Lgl; also known as L(2)gl], is an evolutionarily conserved protein that was discovered in the vinegar fly *Drosophila*, where its depletion results in tissue overgrowth and loss of cell polarity. Lgl links cell polarity and tissue growth through regulation of the Notch and the Hippo signalling pathways. Lgl regulates the Notch pathway by inhibiting V-ATPase activity via Vap33. How Lgl regulates the Hippo pathway was unclear. In this current study, we show that V-ATPase activity inhibits the Hippo pathway, whereas Vap33 acts to activate Hippo signalling. Vap33 physically and genetically interacts with the actin cytoskeletal regulators RtGEF (Pix) and Git, which also bind to the Hippo protein (Hpo) and are involved in the activation of the Hippo pathway. Additionally, we show that the ADP ribosylation factor Arf79F (Arf1), which is a Hpo interactor, is involved in the inhibition of the Hippo pathway. Altogether, our data suggest that Lgl acts via Vap33 to activate the Hippo pathway by a dual mechanism: (1) through interaction with RtGEF, Git and Arf79F, and (2) through interaction and inhibition of the V-ATPase, thereby controlling epithelial tissue growth.

## Teaser

**Summary:** The *Drosophila* tumour suppressor Lgl and Vap33 activate the Hippo pathway by a dual mechanism, involving RtGEF, Git and Arf79F, and the inhibition of the V-ATPase.

## INTRODUCTION

Deregulation of cell polarity and the epithelial-to-mesenchymal transition are hallmarks of human cancer (Hanahan and Weinberg, 2011; Muthuswamy and Xue, 2012; Stephens et al., 2018). Key regulators of cell polarity, Lethal (2) giant larvae [Lgl; also known as L(2)gl], Scribble (Scrib) and Discs large (Dlg; also known as Dlg1), were discovered in *Drosophila* as neoplastic tumour suppressors; these proteins regulate apical-basal cell polarity via their antagonistic interactions with the Par–aPKC and Crumbs (Crb) polarity complexes, and also limit cell proliferation (Stephens et al., 2018). Lgl functions to antagonise the activity of atypical protein kinase C (aPKC), and conversely aPKC phosphorylates and inhibits Lgl, in cell polarity regulation and tissue growth control (Stephens et al., 2018). In *Drosophila*, Lgl–aPKC plays a role in the control of tissue growth (cell proliferation and survival) that is distinct from their role in cell polarity regulation; *lgl* mutation or aPKC activation in eye epithelial tissue causes increased cell proliferation and survival without loss of apico-basal cell polarity (Grzeschik et al., 2007, 2010). Thus, the Lgl–aPKC axis regulates tissue growth independently of cell polarity effects.

The human Lgl orthologue, LLGL1, has a conserved function with *Drosophila* Lgl, as its expression in *Drosophila* rescues the tumorigenic defects of *lgl* mutants (Grifoni et al., 2004; Grzeschik et al., 2010). Reduced expression or mutations in LLGL1 (also known as HUGL1), are associated with hepatocellular carcinoma (Lu et al., 2009), malignant melanoma (Kuphal et al., 2006) and colorectal cancer (Schimanski et al., 2005). Similarly, aberrant localization or deletion of the second human Lgl homolog, LLGL2 (also known as HUGL2), is associated with gastric epithelial dysplasia and adenocarcinoma (Lisovsky et al., 2009), and with pancreatic intraepithelial neoplasia and ductal adenocarcinoma (Lisovsky et al., 2010). Mislocalization of LLGL1 and/or LLGL2 is also observed in other human cancers, including lung adenocarcinoma (Imamura et al., 2013) and ovarian cancer (Grifoni et al., 2007). As occurs in *Drosophila*, the mislocalization or dysfunction of LLGL1 and/or LLGL2 in cancer is also associated with altered aPKC localization or activity (Grifoni et al., 2007; Imamura et al., 2013).

We previously made the novel discovery that in *Drosophila* Lgl acts independently of its apico-basal cell polarity role to regulate the Salvador–Warts–Hippo (Hippo) negative tissue growth control pathway (Grzeschik et al., 2007, 2010). The core of the Hippo pathway involves the serine-threonine protein kinases Hippo (Hpo) and Warts (Wts), which respond to cell–cell contact and tissue architectural cues to control tissue growth via phosphorylating the co-transcriptional activator Yorkie (Yki), which regulates cell proliferation genes (e.g. Cyclin E) and cell survival genes (e.g. Diap1) (Grusche et al., 2010; Richardson and Portela, 2017). Lgl depletion or aPKC activation (which inhibits Lgl) impairs the Hippo pathway via mislocalization of the Hpo protein, away from the apical cortex, where it is normally activated by apical cues (Grzeschik et al., 2010; Parsons et al., 2014a). Thus, Lgl–aPKC controls the Hippo pathway by regulating Hpo localization and activity. In mammalian systems, deregulation of Lgl–aPKC impairs Hippo signalling and induces cell transformation, which mechanistically involves the association of aPKC with the Hpo orthologues, MST1 and MST2 (also known as STK4 and STK3, respectively), thereby uncoupling MST from the downstream kinases LATS1 and LATS2 (Wts in flies) and leading to increased nuclear YAP (Yki) activity (Archibald et al., 2015), consistent with what we observe in *Drosophila* (Grzeschik et al., 2010).

We have also shown that in *Drosophila* Lgl plays a novel regulatory role in ligand-dependent Notch signalling (Parsons et al., 2014b; Portela et al., 2015). Notch activation depends on the cleavage by Adam proteases to produce Notch^ext^, and then processing by γ-secretase to produce the active form Notch^ICD^ (where ICD refers to the intracellular domain), which translocates to the nucleus to activate transcription of target genes, such as the *E(spl)* complex, as well as the cell proliferation and survival genes (Ntziachristos et al., 2014). We found that intracellular Notch accumulated in *lgl* mutant tissue, resulting in elevated Notch signalling that contributes to the overgrowth defects in *lgl* mutant tissue (Grzeschik et al., 2010; Parsons et al., 2014b; Portela et al., 2015)*.* In mouse neural development, *Lgl1* knockout causes elevated Notch signalling, which is associated with hyperproliferation and differentiation defects (Klezovitch et al., 2004), whereas in zebrafish, *Lgl1* knockdown in the developing retina leads to elevated Notch signalling and neurogenesis defects, which are rescued by blocking Notch activity (Clark et al., 2012).

Intriguingly, we recently found that *lgl* mutant tissue exhibited increased LysoTracker incorporation (Parsons et al., 2014b; Portela et al., 2015), indicating that there is elevated vesicle acidification due to the activity of the Vacuolar-ATPase (V-ATPase). In the Notch signalling pathway, γ-secretase activity is dependent on vesicle acidification regulated by V-ATPase activity (Kobia et al., 2014; Vaccari et al., 2010). Therefore, in *lgl* mutant tissue the increased V-ATPase activity elevates γ-secretase activity and Notch cleavage, forming Notch^ICD^, and leading to elevated Notch target gene expression. Consistent with this, genetically or chemically reducing vesicle acidification or V-ATPase function reduces the elevated Notch signalling in *lgl* mutant tissue (Parsons et al., 2014b; Portela et al., 2015). Thus, elevated Notch signalling in *lgl* mutant tissue is due to increased vesicle acidification and elevated γ-secretase activity.

To identify novel proteins that link Lgl to the V-ATPase, we undertook affinity purification-mass spectrometry (AP-MS) analysis of Lgl in *Drosophila* S2 tissue culture cells (Portela et al., 2018). Lgl interacted with aPKC and Par6 with high significance as expected. Among the novel interactors of Lgl that bound at high significance, the standout protein involved in endocytosis was the VAMP-(v-SNARE)-associated protein, Vap33, which is an orthologue of human VAPA and VAPB (Portela et al., 2018). Vap33 (VAPA/B) physically and genetically interacts with endocytic regulators and is involved in endo-lysosomal trafficking, with mutations in *Drosophila Vap33* and human *VAPB* resulting in endocytic defects, including the accumulation of the early endosome Rab5 marker (Sanhueza et al., 2015), a phenotype we also observed in *Drosophila lgl* mutant tissue (Parsons et al., 2014b; Portela et al., 2015). We confirmed the binding of Vap33 to Lgl by co-immunoprecipitations (co-IPs) from S2 cells and *in vivo* in *Drosophila* epithelial cells (Portela et al., 2018) by using proximity ligation assays (PLAs) (Soderberg et al., 2006). Comparison of our data with the global *Drosophila* proteomics network has revealed that Vap33 and Lgl form a network with V-ATPase subunit proteins (Guruharsha et al., 2011; Portela et al., 2018). Interaction with human VAPA, VAPB and V-ATPase proteins is also evident in the human proteome (Huttlin et al., 2015; Portela et al., 2018). We also find that *Vap33* overexpression rescues the elevated Notch signalling in *lgl* mutant eye epithelial clones and the *lgl* mutant adult eye phenotype, whereas knockdown of *Vap33* enhances these eye defects (Portela et al., 2018). *Vap33* overexpression also reduces V-ATPase activity, as assayed by LysoTracker levels (Petzoldt et al., 2013; Portela et al., 2018). Moreover, in *lgl*-knockdown S2 cells and in *lgl* mutant tissue, the interaction between Vap33 and V-ATPase components is decreased (Portela et al., 2018). Thus, Lgl binds to and facilitates the binding of Vap33 to V-ATPase components, which inhibits V-ATPase activity, thereby controlling vesicle acidity, γ-secretase activity and Notch signalling*.*

Although our previous studies have dissected how Lgl regulates the Notch pathway, it is currently not known precisely how Lgl regulates the Hippo pathway. Thus, in this study, we focused on the mechanism of this regulation. We show that the V-ATPase inhibits the Hippo pathway and conversely that Vap33 activates the Hippo pathway. Mechanistically, Vap33 is connected to the Hippo pathway by interacting with the cytoskeletal regulators, RtGEF (Pix), Git and Arf79F (Arf1), which bind to the Hpo protein kinase. Our findings are consistent with a model whereby Lgl–Vap33 promotes Hippo signalling via a dual mechanism through interaction with RtGEF, Git and Arf79F, and by inhibiting the V-ATPase.

## RESULTS

### The Hippo signalling pathway is negatively regulated by V-ATPase activity in *Drosophila*

**Fig. 1.**
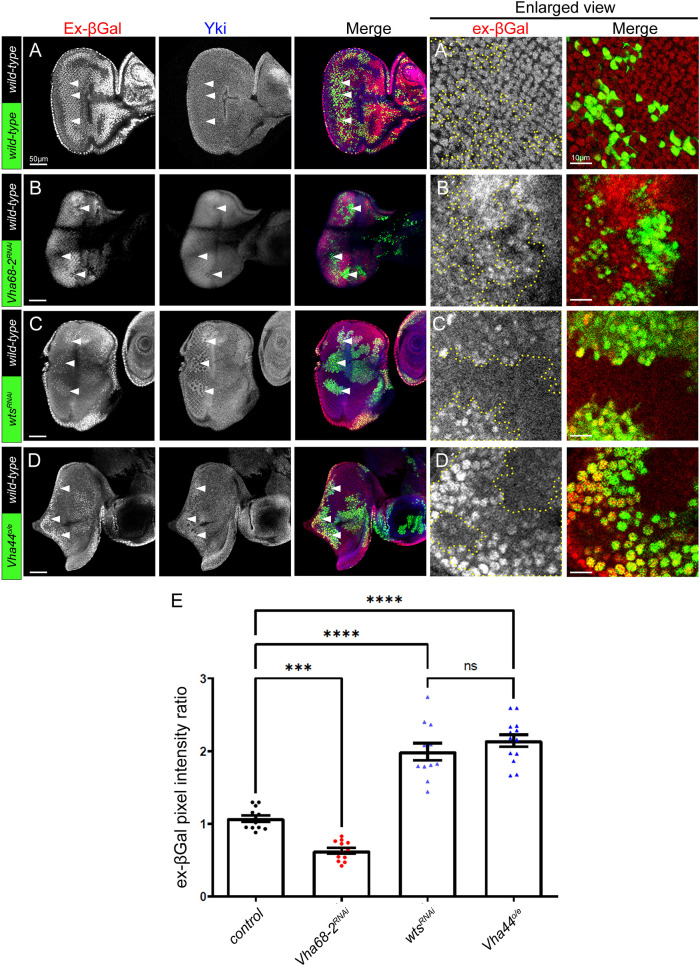
**The Hippo signalling pathway is negatively regulated by V-ATPase activity in *Drosophila*.** (A–D) Confocal planar sections of mosaic eye discs containing the Yki target reporter *ex-lacZ*, stained for βGal (grey; red in merges), and for Yki (grey; blue in merges). Example GFP-positive clones are indicated by arrowheads. (A) Example of a control mosaic disc showing endogenous expression of *ex-lacZ* and Yki in the eye epithelium. (B) Example of a *Vha68-2^RNAi^* mosaic disc, with RNAi-expressing clones being GFP-positive. *Vha68-2* knockdown leads to downregulation of the Yki target, *ex-lacZ* (βGal) reporter and Yki levels. (C) Example of a *wts^RNAi^* mosaic disc with RNAi-expressing clones being GFP-positive. *wts* knockdown leads to upregulation of the Yki target, *ex-lacZ* (βGal) reporter and Yki levels. (D) Example of a *Vha44* overexpression mosaic disc, with Vha44 overexpressing clones being GFP-positive. *Vha44* overexpression leads to upregulation of the Yki target, *ex-lacZ* (βGal) reporter and Yki levels. (A′,B′,C′,D′) Higher magnification examples of *ex-lacZ* (βGal) stainings in GFP-positive clones (areas outlined by yellow dotted line) for the different samples. (E) Quantification of the *ex-lacZ* (βGal) pixel intensity ratio of the transgenic clones compared to wild-type clones (*n*=11–14). Error bars represent s.e.m. ****P<0.0001; ***P=0.0009; ns, not significant (one-way ANOVA with Bonferroni post-test). Scale bars: 50 μm (A–D); 10 μm (A′–D′). Posterior is to the left in all images.

Our previous studies have revealed the involvement of Lgl in the negative regulation of the V-ATPase (Portela et al., 2018), which is an important regulator of the Notch pathway (Kobia et al., 2014; Vaccari et al., 2010). Given that the *lgl* mutant adult eye phenotype (which is due to both Notch and Hippo pathway deregulation) is suppressed by reducing V-ATPase levels [more so than individually inhibiting Notch signalling or reducing Yki and Scalloped (Sd, a TEAD family transcription factor) activity (Grzeschik et al., 2010; Parsons et al., 2014b; Portela et al., 2015)], we suspected that the V-ATPase activity might be involved in the regulation of the Hippo pathway. The V-ATPase comprises several subunits, and knockdown of any of these proteins reduces V-ATPase function (Collins and Forgac, 2020; Dow, 1999; Dow et al., 1997; Eaton et al., 2021). Indeed, reducing V-ATPase levels and activity by using a *Vha68-2* RNAi line resulted in higher Hippo pathway activity as assessed by reduced Yki nuclear staining, and lower expression of the Yki target, *expanded (ex)-lacZ* (*expanded* gene promoter driving expression of lacZ) (Fig. 1B) compared to the wild-type control (Fig. 1A, quantified in E). Conversely, knockdown of the core Hippo pathway gene, *wts*, resulted in impaired Hippo pathway signalling, as assessed by increased Yki nuclear staining and Yki target gene expression (*ex-lacZ*) (Fig. 1C), and a similar level of pathway impairment was observed upon activating the V-ATPase by overexpressing another V-ATPase subunit, namely Vha44 (Petzoldt et al., 2013) (Fig. 1D, quantified in E), which has been previously shown to elevate V-ATPase activity (Petzoldt et al., 2013). Overexpression of *Vha44* has been previously shown to activate the JNK signalling pathway; however, in the eye epithelium JNK activity does not block Hippo signalling but instead is involved in activating Hippo (Doggett et al., 2011; La Marca and Richardson, 2020). Thus, the effect observed upon *Vha44* overexpression on the elevation of the Yki target, *ex-lacZ*, occurs in the background of the elevated JNK-mediated inhibition of Yki activity. From these results we conclude that the V-ATPase negatively regulates the Hippo signalling pathway, leading to the upregulation of Yki activity.

### Vap33 activates the Hippo pathway

**Fig. 2.**
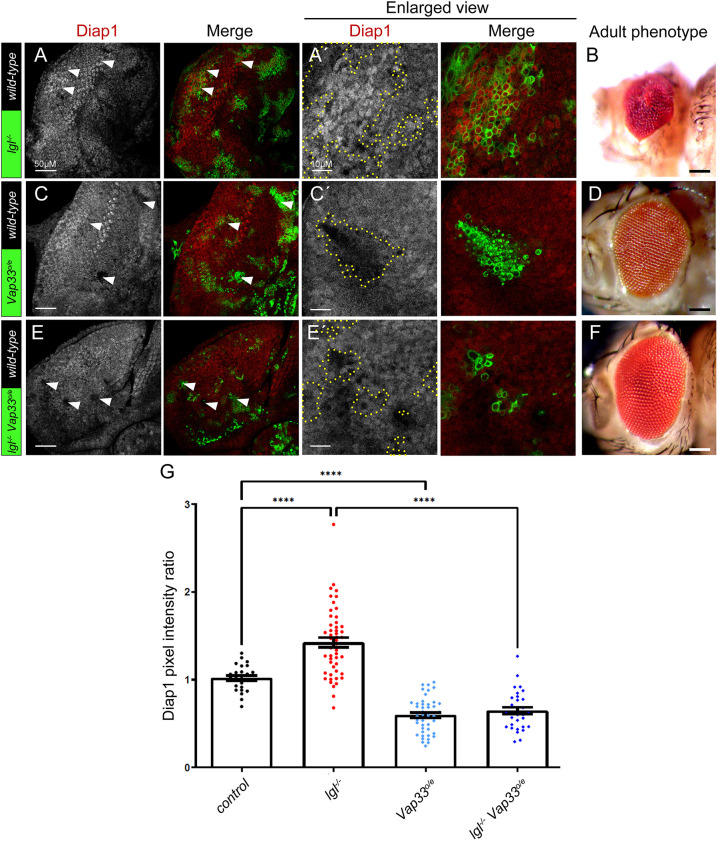
**Vap33 activates the Hippo pathway.** (A,C,E) Confocal planar sections of mosaic eye discs, stained for the Yki target Diap1 (grey; red in merges). Mutant clones are GFP positive, and examples are indicated by arrowheads. A′, C′ and E′ show higher magnifications of Diap1 stainings for the different mosaic tissues (areas outlined by yellow dotted line). (A) Example of a *lgl*^−^ mosaic disc showing elevated Diap1 levels in the mutant clones. (B) *lgl*^−^ mosaic adult female eye, showing a distorted disorganized eye phenotype. (C) Example of *Vap33^o/e^* mosaic disc showing reduced Diap1 levels. (D) *Vap33^o/e^* mosaic adult female eye, showing slight disorganization in the arrangement of ommatidia. (E) Example of *lgl*^−^ *Vap33^o/e^* mosaic disc showing normalized Diap1 levels. (F) *lgl*^−^ *Vap33^o/e^* mosaic adult female eye, showing only slight disorganization of the ommatidial arrangement. Eye images are representative of ∼20 animals examined. (G) Quantification of Diap1 pixel intensity ratio of mutant/transgenic clones compared with wild-type clones (*n*=22–48). Error bars represent s.e.m. ****P<0.0001 (one-way ANOVA with Bonferroni post-test). Scale bars: 50 μm (A,C,E); 10 μm (A′,C′,E′); 100 μm (B,D,F). Posterior is to the left in all images.

Given that we have previously shown that Lgl functions with Vap33 in negatively regulating the V-ATPase (Portela et al., 2018), we then tested whether Vap33 is also involved in Hippo pathway regulation. Here, we used the canonical Yki target, Diap1 (Harvey and Tapon, 2007), to assess Hippo pathway activity. Although Jak-STAT signalling has been shown to induce Diap1 expression in the wing disc during development (Recasens-Alvarez et al., 2017), expression profiling after activation of the Jak-STAT signalling in the eye epithelium did not identify Diap1 as a target (Flaherty et al., 2009). Additionally, there are no reports that Lgl depletion in eye disc clones elevates Jak-STAT signalling (Stephens et al., 2018), but instead loss of cell polarity in mutant clones of the apico-basal polarity gene, *scrib*, in the eye disc results in expression of the Jak-STAT pathway ligand Upd and non-cell autonomous induction of Jak-STAT signalling in the surrounding wild-type cells (Bunker et al., 2015; Fahey-Lozano et al., 2019; Schroeder et al., 2013). Thus, Diap1 expression is a reliable reporter of Hippo pathway activity in the eye epithelium. As we have previously reported (Grzeschik et al., 2010), in *lgl* mutant clones the levels of the Hippo pathway target protein Diap1 were increased (Fig. 2A, quantified in G) and a distorted adult eye phenotype was observed (Fig. 2B). We found that when Vap33 was overexpressed in clones, the expression of Diap1 was reduced (Fig. 2C, quantified in G) and adult eyes appeared only slightly disorganized (Fig. 2D), consistent with Yki activity being reduced. Importantly, overexpression of *Vap33* in *lgl* mutant clones resulted in a reduction of the elevated Diap1 protein levels (Fig. 2E) that were observed in *lgl* mutant clones (Fig. 2A) to below wild-type levels, similar to what is seen upon *Vap33* overexpression alone (Fig. 2C, quantified in Fig. 2G), and rescued the *lgl* mutant mosaic distorted adult eye phenotype towards that of the Vap33 overexpressing mosaic adult eye (Fig. 2F compared with B and D), indicating that Vap33 is epistatic to Lgl. Taken together, these results show that Vap33 activates the Hippo pathway and that Vap33 is epistatic to *lgl* impairment in the activation of the Hippo pathway.

### Vap33 and Lgl form a protein interaction network with RtGEF, Git, Arf79F and Hpo

Given that our results implicated Vap33 in the regulation of the Hippo pathway, we sought to determine whether this occurs via protein–protein interactions. Interestingly, the global human proteomics analysis has elucidated the protein interaction network of the human Vap33 orthologues VAPA and VAPB, and revealed amongst the interacting proteins the Hpo orthologues MST1 and MST2 (Huttlin et al., 2015). To determine whether *Drosophila* Vap33 also interacts with Hpo we conducted affinity purification-mass spectrometry (AP-MS) on endogenously expressed Vap33–YFP *in vivo* using immunoprecipitation with a GFP nanobody (Neumuller et al., 2012). We decided to use an *in vivo* approach because the common *Drosophila* cell line (S2 cells, thought to be derived from haemocytes) used for protein interaction analysis are not polarized cells, and by using *Drosophila* tissues, we hoped to reveal physiologically relevant interactors important in polarized epithelial tissues. We prepared proteins from Vap33–YFP-expressing embryos and conducted the experiment in triplicate together with controls. As expected, among the AP-MS Vap33 interactors, we observed the V-ATPase component Vha68-2 as a medium confidence interactor [significance analysis of interactome (SAINT) score ∼0.77] and three other V-ATPase components as lower confidence interactors (Table S3). Surprisingly, Lgl and Hpo were not detected, suggesting that under the conditions used in embryonic cells these proteins do not form strong interactions with Vap33. However, pertinently, with respect to the Hippo pathway, we detected as high confident interactors (SAINT scores ∼1), the Hpo-interacting proteins, RtGEF (also known as Pix; a Rho-type guanine nucleotide exchange factor) and Git (G protein-coupled receptor kinase interacting ArfGAP), are actin cytoskeletal regulators that form a protein complex (Frank and Hansen, 2008; Zhou et al., 2016), and also bind to and activate Hpo (Dent et al., 2015).

**Fig. 3.**
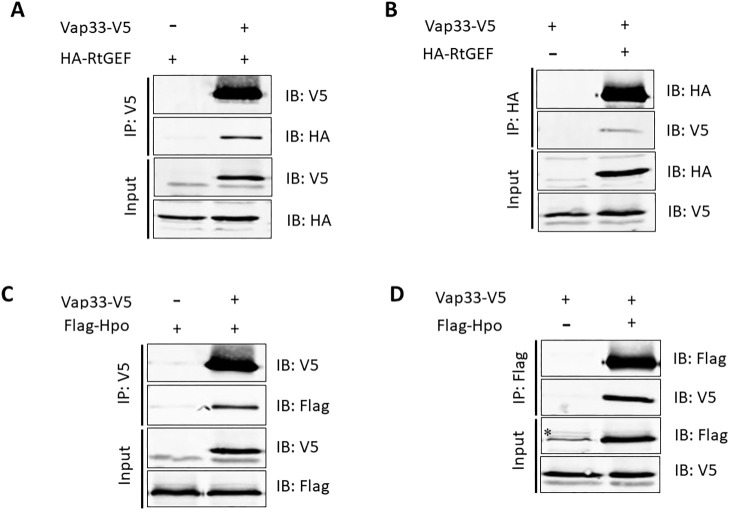
**Vap33 interacts with RtGEF and Hpo in S2 cells.** (A–D) The indicated protein constructs were expressed in S2 cells, and protein interactions were analysed by co-immunoprecipitation (co-IP). IP: immunoprecipitation antibody; IB: immunoblot antibody. Asterisk in D indicates non-specific band. Images are representative of two experimental repeats.

To confirm these interactions, we conducted co-immunoprecipitation (co-IP) analyses in S2 cells. We co-transfected Vap33 tagged with V5 and RtGEF tagged with HA and conducted IP-western blot analysis (Fig. 3). Vap33–V5 co-immunoprecipitated with RtGEF–HA in both directions (Fig. 3A,B). We also investigated whether Vap33 and Hpo could form a complex by co-transfecting Vap33–V5 and Hpo–Flag in S2 cells and conducting IP followed by western blotting. Indeed, Vap33 co-immunoprecipitated with Hpo in both directions (Fig. 3C,D). Thus, Vap33 binds to both RtGEF and Hpo, supporting our AP-MS data, and consistent with previous studies showing that Hpo interacts with RtGEF and Git (Dent et al., 2015), and that the human Vap33 orthologues VAPA and VAPB interact with the Hpo orthologues MST1 and MST2 (Huttlin et al., 2015).

**Fig. 4.**
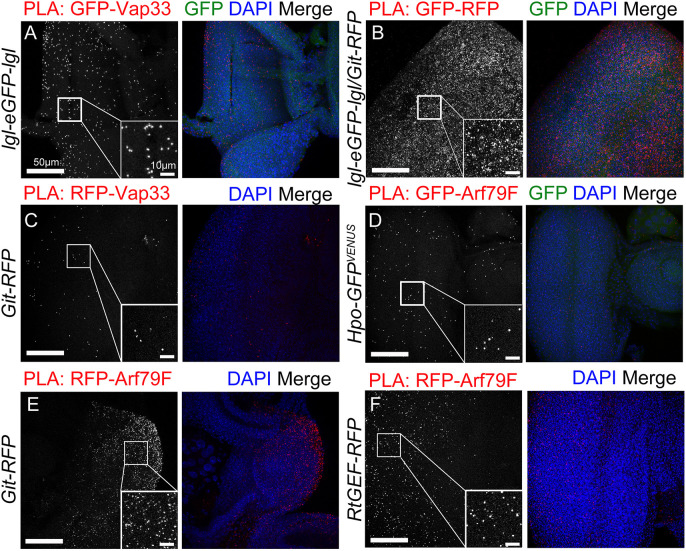
**Vap33 interacts with Git, Arf79F interacts with Git, RtGEF and Hpo, and Lgl interacts with Vap33 and Git *in vivo*.** (A–F) Confocal planar images showing *in situ* proximity ligation assays (PLAs) in third-instar larval eye discs. Positive PLA results between the indicated proteins are visualized by punctate signal (grey; red in the merges). Nuclei are stained with DAPI (blue). Insets show high magnification images of the PLA foci. (A) Positive-control PLA in *lgl-eGFP-lgl* eye discs using antibodies against GFP and Vap33. (B) PLA in *lgl-eGFP-lgl*/*Git-RFP* eye discs using antibodies against RFP and GFP. (C) PLA in *Git-RFP* eye discs using antibodies against RFP and Vap33. (D) PLA in *Hpo-GFP* eye discs using antibodies against GFP and Arf79F. (E) PLA in *Git-RFP* eye discs using antibodies against RFP and Arf79F. (F) PLA in *RtGEF-RFP* eye discs using antibodies against RFP and Arf79F. Images are representative of two or three experimental repeats. Scale bars: 50 μm.

We then examined whether interactions between Lgl or Vap33 and RtGEF and/or Git occurred in *Drosophila* larval epithelial tissues, by using the *in situ* PLAs (Soderberg et al., 2006). We have previously used the PLA method to detect robust interactions between Lgl and Vap33 in *Drosophila* epithelial tissue, whereas controls lacking expression of one protein or without one of the antibodies showed minimal positive foci (Portela et al., 2018). First, using larval tissues (eye-antennal epithelial, salivary gland and brain) from endogenously GFP-tagged Lgl flies (Portela et al., 2018; Venken et al., 2011) and antibodies against GFP and Vap33, we confirmed that PLA foci were observed for Lgl and Vap33 (Fig. 4A; Fig. S1G,H) (Portela et al., 2018), suggesting that these proteins physically interact *in vivo*. We also observed foci for Lgl–GFP and atypical protein kinase C (aPKC), by using GFP and aPKC antibodies in eye-antennal discs and salivary gland cells (Fig. S1A,C). Importantly, no PLA signals were observed in the single antibody negative controls (Fig. S1B,D–F). We then conducted PLAs on eye-antennal epithelial and salivary gland tissues using flies expressing the endogenously tagged Lgl–GFP and Git–RFP transgene (Dent et al., 2019), using anti-GFP and -RFP antibodies, which revealed multiple PLA foci (Fig. 4B; Fig. S1J). Likewise, PLAs performed on Git–RFP transgenic fly eye-antennal epithelial and salivary gland tissues using anti-RFP and -Vap33 antibodies, revealed multiple PLA foci (Fig. 4C; Fig. S1K). Altogether, the PLA data confirm that Git interacts *in vivo* with Vap33 and Lgl in the *Drosophila* larval eye-antennal epithelium (Fig. 4), as well as in other polarized cells in the salivary gland and brain (Fig. S1).

Interestingly, in mammalian cells, Git interacts with the ADP ribosylation factor Arf1 and inhibits its activity (Zhou et al., 2016), and examination of the *Drosophila* Hippo pathway proteomics dataset (Kwon et al., 2013) revealed that the *Drosophila* Arf1 ortholog Arf79F is a high confidence interactor with Hpo (SAINT score >0.8), which was validated by co-IP analysis (Kwon et al., 2013). Based on these physical interactions, we also tested by PLA whether Arf79F interacted with Git, RtGEF and Hpo. Indeed, using flies containing a Hpo–GFP–Venus transgene (Pojer et al., 2021) and antibodies against Arf79F, we confirmed via PLA that Hpo and Arf79F interact in eye-antennal epithelial and brain tissue (Fig. 4D; Fig. S1I). Similarly, using PLAs on eye-antennal discs and salivary glands from Git–RFP or RtGEF–RFP transgenic flies (Dent et al., 2019) and anti-RFP and -Arf79F antibodies, Arf79F was confirmed to interact with Git and RtGEF (Fig. 4E,F; Fig. S1L,M). Thus, we have revealed that Arf79F binds to Git, RtGEF and Hpo in eye-antennal tissue (Fig. 4), as well as salivary gland or brain tissues (Fig. S1). The lower levels of PLA signals in some samples might be due to low levels of expression of the proteins or lower levels of protein–protein interactions.

### Vap33 overexpression rescues increased Hippo pathway target gene expression in *RtGEF* mutant tissue

**Fig. 5.**
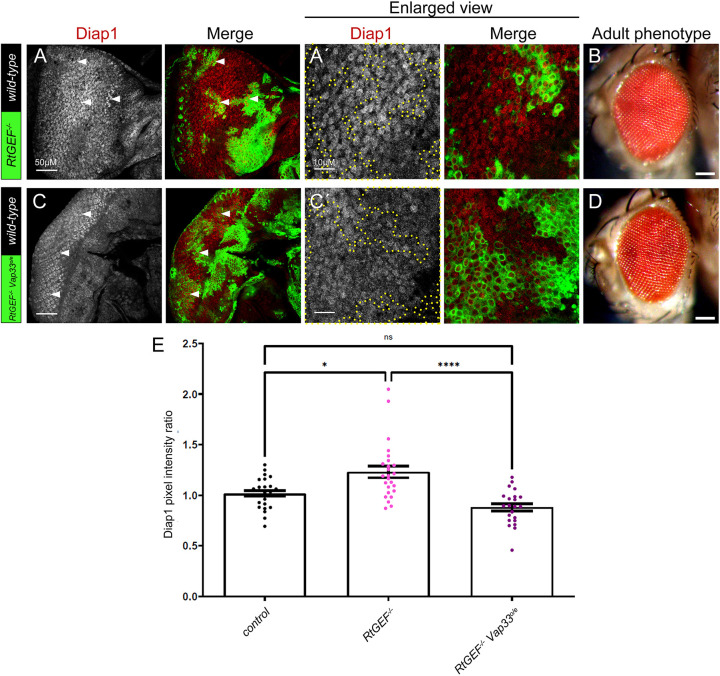
**Vap33 overexpression rescues increased Hippo pathway target gene expression in *RtGEF* mutant tissue.** (A) Confocal planar section of a *RtGEF^−^* mosaic disc stained for the Yki target Diap1 (grey; red in merges, mutant clones are GFP-positive, examples indicated by arrowheads). (B) *RtGEF^−^* mosaic adult female eye showing a mostly wild-type appearance. (C) Confocal planar section of *RtGEF^−^ Vap33^o/e^* mosaic disc stained for Diap1 (grey, or red in merge, mutant tissue is GFP-positive, examples indicated by arrowheads). o/e, over expressing. (D) *RtGEF^−^ Vap33^o/e^* mosaic adult female eye showing slight disorganization of the ommatidial arrangement, similar to what is seen in *Vap33^o/e^* mosaic eyes (Fig. 2D). A′ and C′ are higher magnifications of Diap1 stainings of the respective samples (areas of clones are outlined by yellow dotted line). Eye images are representative of ∼20 animals examined. (E) Quantification of Diap1 pixel intensity ratio of mutant or transgenic clones compared to wild-type clones (*n*=22–25). Error bars represent s.e.m. ****P<0.0001; *P=0.019; n.s., not significant (one-way ANOVA with Bonferroni post-test). Scale bars: 50 μm (A,C); 10 μm (A′,C′); 100 μm (B,D). Posterior is to the left.

To determine whether *Vap33* genetically interacts with *RtGEF*, we examined the expression of the Hippo pathway target Diap1 in *RtGEF* mutant (*RtGEF^P1036^*) clones that overexpress Vap33. Individually, *RtGEF* mutant clones showed increased Diap1 expression (Fig. 5A), as expected (Dent et al., 2015), and resulted in a slightly enlarged adult eye phenotype (Fig. 5B). The elevated Diap1 expression observed in *RtGEF* mutant clones was rescued to normal levels upon *Vap33* overexpression (Fig. 5C, quantified in E), and the adult eye size was normalized (Fig. 5D). Thus, consistent with the protein interactions, RtGEF and Vap33 genetically interact in the regulation of the Hippo pathway in a manner consistent with both Vap33 and RtGEF acting to induce Hippo pathway activity.

### Git knockdown rescues the reduced Hippo pathway target gene expression in *Vha68-2* mutant clones

**Fig. 6.**
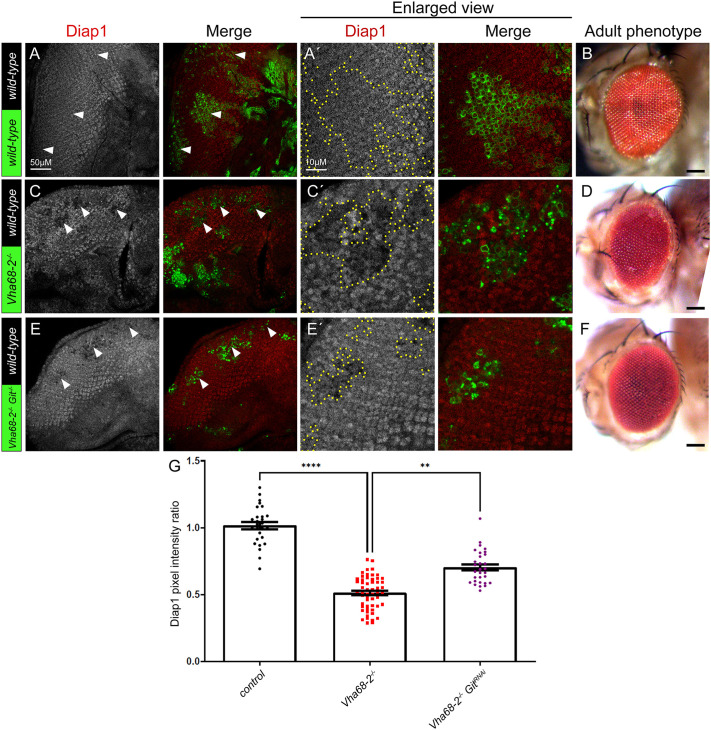
***Git* knockdown rescues the reduced Hippo pathway target gene expression in *Vha68-2* mutant clones.** (A) Confocal planar section of control mosaic eye discs (clones marked with GFP) stained for the Yki target Diap1 (grey; red in merges, example GFP-positive clones indicated by arrowheads) showing endogenous expression of Diap1. (B) Control mosaic adult female eye. (C) Confocal planar section of *Vha68-2^−/^*^−^ mosaic disc stained for Diap1 (grey, or red in merges, mutant clones are GFP-positive, examples indicated by arrowheads). (D) *Vha68-2^−/^*^−^mosaic adult female eye showing disorganization of the ommatidial arrangement. (E) Confocal planar section of *Vha68-2^−/^*^−^ *Git^RNAi^* mosaic disc stained for Diap1 (grey, or red in merge, mutant tissue is GFP-positive, examples indicated by arrowheads). (F) *Vha68-2^−/^*^−^ *Git^RNAi^* mosaic adult female eye showing a mostly normal phenotype. (A′,C′,E′) Higher magnifications of Diap1 stainings of the corresponding samples. Eye images are representative of ∼20 animals examined. (G) Quantification of Diap1 pixel intensity ratio of the mutant or transgenic clones relative to wild-type clones (*n*=30–55). Error bars represent s.e.m. ****P<0.0001; **P=0.054 (one-way ANOVA with Bonferroni post-test). Scale bars: 50 μm (A,C,E); 10 μm (A′,C′,E′); 100 μm (B,D,F). In all images, posterior is to the left.

Next, we examined genetic interactions between Git and a component of the V-ATPase, Vha68-2, in Hippo pathway regulation. Previous analysis of Git and RtGEF knockdown tissue had revealed that Yki targets are upregulated; however, the adult mosaic eyes were similar to the wild-type control (Dent et al., 2015; data not shown). As we have previously observed, *Vha68-2* mutant clones are small although they do not show indications of cell death and are retained into pupal development (Portela et al., 2018). Consistent with our analysis of *ex-lacZ* (Fig. 1C), *Vha68-2* mutant clones also showed reduced expression of the Hippo pathway target Diap1 (Fig. 6C) relative to wild-type clones (Fig. 6A, quantified in G), and resulted in reduced and disorganized adult eyes relative to the wild-type control (Fig. 6D compared with B). When Git was knocked down using a transgenic RNAi line in *Vha68-2* mutant clones, partial rescue of the reduced Diap1 expression was observed (Fig. 6E, quantified in G) and the disorganized *Vha68-2* mutant mosaic adult eye phenotype was strongly rescued resulting in a phenotype that was similar to wild-type adult eyes (Fig. 6F compared with D and B). These results show that the reduced Yki activity (low Diap1 expression) that occurs in Vha68-2 mutant clones is rescued by Git1 knockdown and suggest that the activation of Hippo signalling (reduced Diap1 expression) that occurs upon reducing V-ATPase activity depends upon Git. A possible interpretation for this genetic interaction is that V-ATPase activity might oppose the action of Git1 and/or RtGEF to activate Hpo.

### Arf79F is required for Lgl-mediated regulation of the Hippo pathway

**Fig. 7.**
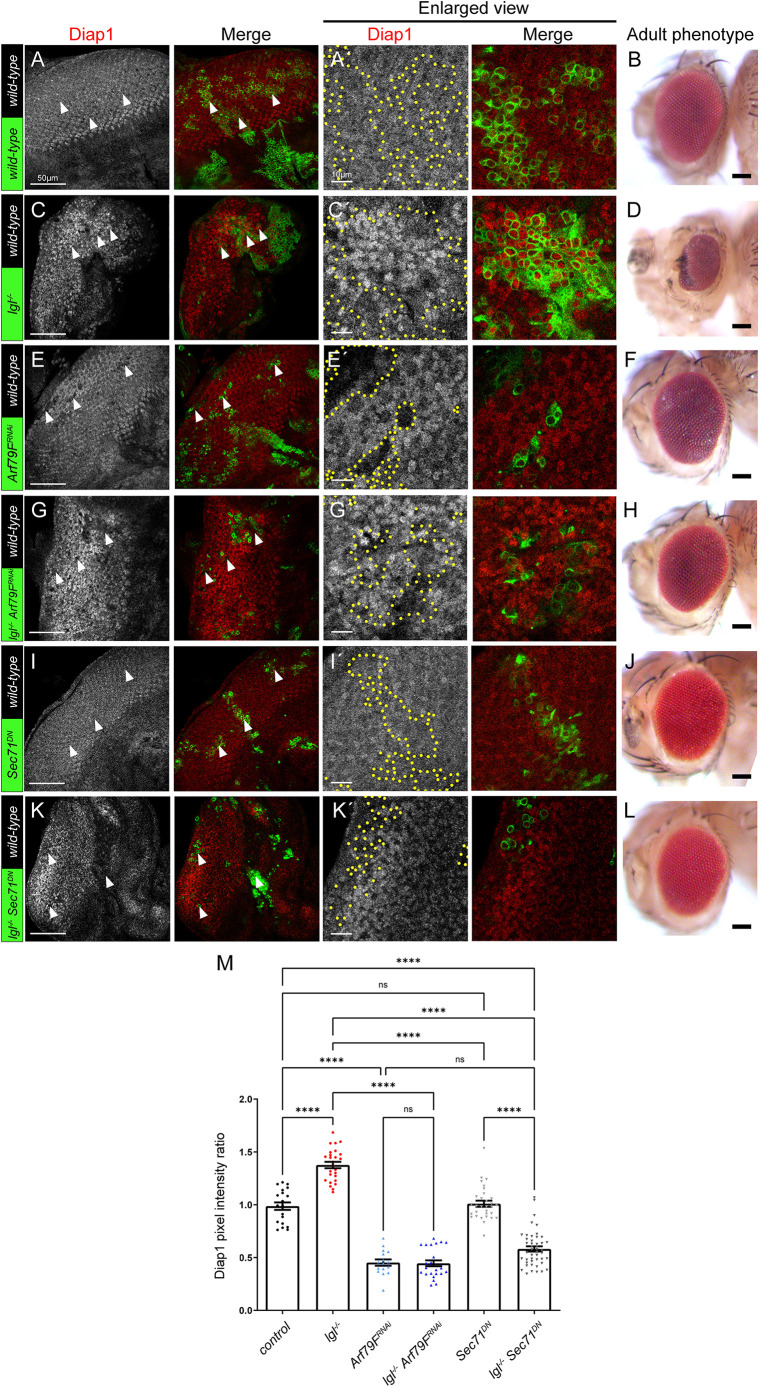
**Knockdown of Arf79F prevents the upregulation of Hippo pathway targets.** (A,C,E,G,I,K) Confocal planar images of mosaic third-instar larval eye-antennal discs, stained for the Yki target, Diap1 (grey; red in merge). In all instances, example GFP-positive clones are marked by arrowheads. (A′,C′,E′,G′,I′,K′) Higher magnifications of Diap1 stainings in the corresponding samples (areas of clones are outlined by yellow dotted line). (A) Control eye discs (clones marked by GFP) showing endogenous expression of Diap1. (B) Control mosaic adult female eye. (C) *lgl*^−^ mosaic disc showing elevated Diap1 expression in mutant clones (GFP positive). (D) *lgl*^−^ mosaic adult female eye showing a distorted eye with disorganized arrangements of ommatidia. (E) *Arf79F^RNAi^* mosaic disc showing reduced Diap1 expression in transgene-expressing clones (GFP positive). (F) *Arf79F^RNAi^* mosaic adult female eye showing slightly disorganized ommatidia arrangements. (G) *lgl*^−^ *Arf79F^RNAi^* mosaic disc showing reduced Diap1 expression in mutant or transgene-expressing clones (GFP positive). (H) *lgl*^−^ *Arf79F^RNAi^* mosaic adult female eye showing a mostly normal eye with only some slightly disorganized ommatidia arrangements. (I) *Sec71^DN^* mosaic disc showing normal Diap1 expression in transgene-expressing clones (GFP positive). (J) *Sec71^DN^* mosaic adult female eye showing slightly disorganized ommatidia arrangements. (K) *lgl*^−^ *Sec71^DN^* mosaic disc showing reduced Diap1 expression in mutant or transgene-expressing clones (GFP positive). (L) *lgl*^−^ *Sec71^DN^* mosaic adult female eye showing a mostly normal eye with only some slightly disorganized ommatidia arrangements. Eye images are representative of ∼20 animals examined. (M) Quantification of Diap1 pixel intensity ratio of mutant or transgene clones relative to wild-type clones (*n*=20–43). Error bars represent s.e.m. ****P<0.0001; n.s., not significant (one-way ANOVA with Bonferroni post-test). Scale bars: 50 μm (A,C,E,G,I,K); 10 μm (A′,C′,E′,G′,I′,K′); 100 μm (B,D,F,H,J,L). Posterior is to the left in all images.

We then tested whether Arf79F is required for the regulation of the Hippo pathway by Lgl. As we previously observed, *lgl* mutant tissue shows impaired Hippo pathway signalling, as revealed by the elevated levels of Diap1 (Fig. 7C compared to the control, A, quantified in M) and results in a distorted, disorganized adult eye phenotype relative to the wild-type control (Fig. 7D compared with B). We therefore sought to determine whether *Arf79F* genetically interacts with *lgl* in its regulation of the Hippo pathway. When *Arf79F* was knocked down, using a RNAi line (which we showed effectively reduced Arf79F protein levels in eye disc clones, Fig. S2A,B), a decrease in Diap1 expression was observed and clones were smaller than wild-type clones (Fig. 7E), suggesting that the *Arf79F* knockdown clones have reduced tissue growth, consistent with Hippo pathway upregulation. However, the *Arf79F* adult mosaic eyes showed only slight disorganization (Fig. 7F compared with the wild-type control in B). Knockdown of *Arf79F* in *lgl* mutant clones also resulted in low Diap1 levels, similar to *Arf79F* knockdown alone (Fig. 7G compared with E, quantified in M), suggesting that *Arf79F* is epistatic to *lgl* in the regulation of the Hippo pathway. Consistent with this, the *lgl* mutant mosaic distorted adult eye phenotype was normalized upon *Arf79F* knockdown (Fig. 7H compared with D and B). Additionally, we used a dominant-negative version of the activator of Arf79F, Sec71 (*Sec71^DN^*) to reduce Arf79F function. When expressed individually in clones, *Sec71^DN^* had no significant effect upon Diap1 levels (Fig. 7I, quantified in M), and did not obviously affect the adult eye phenotype (Fig. 7J). However, when *Sec71^DN^* was expressed in *lgl* mutant clones, lower Diap1 levels were observed (Fig. 7K, quantified in M), and the *lgl* mutant mosaic adult eye phenotype was normalized towards wild-type (Fig. 7L compared with D and B). Interestingly, although *Sec71^DN^* expression did not have a significant effect on Diap1 expression on its own, when expressed in *lgl* mutant clones it reduced Diap1 levels to below that of the control (Fig. 7M), suggesting that Lgl depletion depends on Sec71 (and thus Arf79F) to inhibit Hippo signalling. These results show that, consistent with the binding of Arf79F with Hpo (Fig. 4D), knockdown of Arf79F reduces Diap1 expression, suggesting that Arf79F acts to inhibit the Hippo pathway. Furthermore, knockdown of Arf79F (or expression of *Sec71^DN^*) rescued the elevated expression of Diap1 in *lgl* mutant clones (Fig. 7M), indicating that Arf79F levels/activity are required for the inhibition of Hippo signalling in *lgl* mutant tissue. Thus, Arf79F is a negative regulator of Hippo signalling that acts downstream of Lgl. Since mammalian Git proteins act to inhibit Arf1 activity (Zhou et al., 2016), our results are consistent with a mechanism where Lgl and Vap33 promote RtGEF/Git activity to inhibit Arf79F (Arf1), and thereby activate the Hippo pathway.

## DISCUSSION

**Fig. 8.**
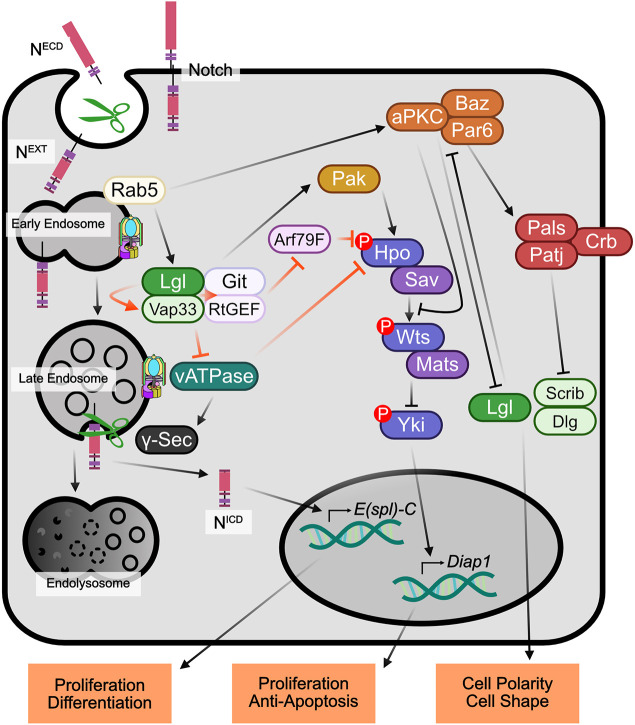
**Model for the regulation of Hippo and Notch signalling by Lgl and aPKC.** Lgl and Vap33 have a dual action in activating Hippo signalling: (1) through binding to RtGEF and Git, which opposes Arf79F inhibition of Hpo, and (2) by binding to V-ATPase components and inhibiting V-ATPase activity, which is an inhibitor of Hippo signalling. The Lgl interactor Rab5 might be involved to link Lgl and Vap33 to early endosomes. It is possible that the RtGEF- and Git-interacting protein Pak kinases (Pak1, Pak3 and Mbt) are involved in activating Hpo downstream of Git and RtGEF. By reducing V-ATPase activity, Lgl and Vap33 inhibits Notch signalling by decreasing the production of the active Notch (N)^ICD^ isoform by γ-Secretase in endosomes, thereby leading to lower expression of Notch targets, such as *E(Spl)-C* genes. Lgl also opposes aPKC activity, and in mammalian cells elevated PKC inhibits Hippo signalling by sequestering Hpo (MST1 and MST2) away from its target Wts (LATS1 and LATS2). Orange arrows indicate the parts of this model that we have provided evidence for in this study.

In this study, we have revealed a new mechanism for the control of the Hippo pathway by the cell polarity regulator Lgl. We show herein that V-ATPase activity leads to inhibition of Hippo signalling, and conversely that Vap33 activates the Hippo pathway downstream of Lgl. We discovered that Vap33 physically interacts with RtGEF and Git using *in vivo* AP-MS. RtGEF and Git are Hpo interactors (Dent et al., 2019), and we confirmed that Vap33 interacts with RtGEF and Hpo in S2 cells, and that Vap33 and Lgl are in close proximity with Git in *Drosophila* cells. We also show that the ADP ribosylation factor Arf79F, the mammalian orthologue of which (ARF1) binds to the mammalian Git orthologues GIT1 and GIT2 (Zhou et al., 2016), and is in close proximity with Git, RtGEF and Hpo in *Drosophila* cells. Functionally, we show that overexpression of Vap33 rescues the elevated Diap1 levels (impaired Hippo signalling) in *RtGEF* mutant clones. Furthermore, the reduced Diap1 expression (elevated Hippo signalling) upon V-ATPase impairment (*Vha68-2* mutant clones) is rescued by knockdown of Git. We also show that Arf79F knockdown leads to reduced Diap1 expression (increased Hippo signalling) downstream of Lgl. Altogether, our data are consistent with a model where Lgl and Vap33 activate the Hippo pathway by a dual mechanism: (1) Lgl and Vap33, through interaction with RtGEF, Git and Arf79F, positively regulate Hippo pathway activity, and (2) Lgl and Vap33, through interaction with components of the V-ATPase, block V-ATPase activation and prevent its negative regulation of the Hippo pathway (Fig. 8). Precisely how the V-ATPase functions to regulate Hippo signalling is unknown. However, the V-ATPase acts to increasing vesicle acidification, and this has been shown to impact the efficacy of many signalling pathways (Collins and Forgac, 2020; Eaton et al., 2021; Pamarthy et al., 2018). The V-ATPase might therefore act to inhibit Hippo pathway signalling by blocking the interaction of Lgl, Vap33, RtGEF, Git and Arf79F with Hpo on endosomes, thereby altering Hpo localization and inhibiting its activity. Consistent with this notion, we previously observed that Lgl colocalizes with endocytic vesicle markers (Parsons et al., 2014b), and that Hpo localization is altered in *lgl* mutant tissue (Grzeschik et al., 2010). Furthermore, Hpo signalling is associated with endosomal regulators (Verghese and Moberg, 2019), and the subcellular localization of Hpo has been shown to be important for its activation, and for Hpo-mediated phosphorylation and activation of its downstream protein kinase Wts (Sun et al., 2015).

In their interactions with the Hippo pathway, Git and RtGEF function to activate Hpo (Dent et al., 2015), and our analyses indicate that Arf79F acts to inhibit Hippo signalling given that the elevated Hippo pathway activity (increased Diap1 expression) in *lgl* mutant clones was normalized upon knockdown or inhibition of Arf79F. Previous studies examining the interactions between mammalian orthologues of Git and Arf79F, have shown that GIT1 and GIT2 act to inactivate ARF1 (Zhou et al., 2016), and therefore it is likely that Git (and RtGEF) act to block Arf79F activity to mediate Hpo activation (Fig. 8). Lgl and Vap33 might then function to promote Git- and RtGEF-mediated inhibition of Arf79F, facilitating activation of Hpo. Arf79F (ARF1) is a regulator of vesicular trafficking (Adarska et al., 2021), and might inhibit Hpo by relocalizing it away from its activators, such as Expanded and Fat, at specific apical subcellular domains (Sun et al., 2015). Interestingly, from our previous Lgl interactome analysis, Arf79F can be linked to Lgl through the Arf79F-binding protein Rab5 (Guruharsha et al., 2011; Kwon et al., 2013), which also binds to Lgl and aPKC at high confidence (SAINT score ∼1) (Portela et al., 2018 and our unpublished data). Thus, it is possible that the early endosomal regulator Rab5 might also be involved in linking Lgl, Vap33 and aPKC to Arf79F in early endosomes, where they could also interact with Vap33, Git, RtGEF and Hpo.

It is unclear whether the RtGEF- and Git-regulated protein kinase Pak (Zhou et al., 2016) is involved in the regulation of Hippo signalling, given that although individual knockdown of two Pak paralogs in *Drosophila*, Pak1 and Pak3, did not affect Hippo pathway signalling (Dent et al., 2015), it is possible that there might be redundancy between Pak1, Pak3 and the third *Drosophila* Pak paralogue Mushroom bodies tiny (Mbt) (Melzer et al., 2013; Menzel et al., 2008, 2007; Schneeberger and Raabe, 2003) in the regulation of Hippo pathway signalling. Pak proteins have been shown to be involved in cell polarity, F-actin regulation and morphogenesis in *Drosophila* (Asano et al., 2009; Baek et al., 2012; Bahri et al., 2010; Conder et al., 2007; Felix et al., 2015; Harden et al., 1996; Menzel et al., 2008, 2007; Schneeberger and Raabe, 2003; Vlachos and Harden, 2011); however, Mbt also plays a role in tissue growth during *Drosophila* developmental (Lim et al., 2019; Melzer et al., 2013), suggesting that Mbt is a candidate for investigation of a potential role downstream of RtGEF and Git in Hippo pathway regulation.

Our analyses here and in our previous study (Portela et al., 2018), have revealed that Lgl interacts with Vap33 to positively regulate the Hippo pathway and to negatively regulate the Notch pathway. Mechanistically, Lgl and Vap33 inhibit the Notch pathway by inhibiting V-ATPase activity and reducing the vesicle acidification that is required for the cleavage of the Notch receptor and release of Notch^ICD^ from vesicles (Portela et al., 2018), where it can then translocate into the nucleus and promote transcription of its target genes (Fig. 8). Interestingly, Arf79F (Arf1) is required for Notch signalling in *Drosophila* haemocyte differentiation, where it promotes Notch trafficking (Khadilkar et al., 2014). Thus, it is possible that Lgl, Vap33, RtGEF and Git might also inhibit Arf79F to reduce Notch signalling, and that in *lgl* mutant tissue elevated Arf79F activity might also contribute to the elevated Notch activation. Indeed, we found that Arf79F is required for the elevated Notch pathway signalling in *lgl* mutant clones, given that *Arf79F* knockdown in *lgl* mutant clones normalized the expression of the Notch pathway reporter, *E(spl)m8-lacZ* (Fig. S3). Whether RtGEF and Git are also regulators of Notch signalling remains to be determined.

In summary, our study has revealed a new mechanism for Hippo pathway regulation by the cell polarity and tumour suppressor protein Lgl. Mechanistically, Lgl and Vap33 function in a dual manner to promote Hippo signalling by both reducing V-ATPase activity, and thereby preventing V-ATPase from inhibiting Hippo signalling and by acting through RtGEF and/or Git to prevent Arf79F from inhibiting Hpo. By promoting Hippo signalling as well as inhibiting Notch signalling, Lgl and Vap33 function to limit tissue growth. Mutation of Lgl therefore results in tissue overgrowth due to inhibition of the Hippo pathway and activation of the Notch pathway. However, despite the dual regulation of Hippo and Notch signalling by Lgl and Vap33, the *lgl* mutant phenotype does not completely mirror the *Vap33* mutant phenotype (Portela et al., 2018), which is likely due to Vap33 having additional roles in vesicle trafficking, proteolysis and signalling (Borgese et al., 2021; Choi et al., 2012; Deivasigamani et al., 2014; Gao et al., 2023; Mao et al., 2019; Sanhueza et al., 2015; Tsuda et al., 2008), and Lgl having additional roles in cell polarity regulation (Grifoni et al., 2013; Stephens et al., 2018). The dual mechanism of Notch and Hippo pathway regulation by Lgl and Vap33 might be important in limiting tissue growth during development and might play a role in tissue homeostasis, enabling cell proliferation to occur after tissue damage, thereby enabling wound repair. Indeed, Hippo pathway signalling is inhibited and Notch pathway signalling is activated after tissue wounding, and both pathways are involved in tissue regeneration (Blanco et al., 2010; Chen et al., 2012; Grusche et al., 2011; Kux and Pitsouli, 2014). However, the involvement of Lgl, Vap33, Git, RtGEF and Arf79F and the V-ATPase in the regulation of Hippo and Notch signalling during the response to tissue wounding remains to be determined. Furthermore, whether the mammalian orthologues of Lgl and Vap33 also act via these mechanisms to control tissue growth in mammals remains to be determined.

## MATERIALS AND METHODS

### *Drosophila* stocks and husbandry

Fly stocks were generated in house or obtained from other laboratories or stocks centres as detailed in Table S1. All *Drosophila* genotypes for all the samples analysed in the figures and detailed in Table S2. All fly stocks and crosses were raised and undertaken on a standard cornmeal, molasses and yeast medium within temperature-controlled incubators kept at 25°C.

### Clonal analysis

Mosaic analysis with a repressible cell marker [MARCM (GFP+); Lee and Luo, 2001) clones were generated as previously described (Grzeschik et al., 2010), using the following stock: *ey-FLP, UAS-GFP; Tub-GAL80, FRT40A; Tub-GAL4/TM6B* MARCM 2L. Crosses were set up and left overnight at room temperature. Adults were then turned daily into new vials and allowed to lay for ∼24 h at 25°C. Third-instar larval (L3) animals were then collected after ∼144 h (6 days after the egg laying period). Samples were collected and prepared as described across multiple days to be similarly aged, and then stored at 4°C in 80% glycerol as necessary prior to mounting.

Heat shock induced Flip-out clones were generated using the stock: *hs-FLP; ex-lacZ; Act>CD2>GAL4, UAS-GFP.* Specifically, clones were induced by heat shocking larvae at 37°C for 15 min at ∼72 h after egg laying. L3 imaginal discs were then dissected and prepared as described 72 h after clone induction.

### Immunofluorescence

L3 eye-antennal discs, were dissected in phosphate-buffered saline (PBS), fixed in 4% paraformaldehyde for 30 min, washed in PBS plus 0.1 or 0.3% Triton X-100 (PBT), and blocked in PBT plus 1% BSA (PBT/BSA). The tissues were incubated with the primary antibody in PBT/BSA overnight at 4°C. After washing off the primary antibodies, the tissues were incubated with the secondary antibodies in PBT for 1 h at room temperature. After washing off the secondary antibodies the samples were mounted in 80% glycerol or Vectashield (Vector Laboratories).

Antibodies used were: mouse β-galactosidase (Sigma, cat. no. G4644, 1:500), rabbit GFP (Invitrogen, cat. no. A11122, 1:500), mouse GFP (Invitrogen, cat. no. A11120, 1:500); mouse RFP (Invitrogen, cat. no. RF5R, 1:100) rabbit Yki (gift from Ken Irvine, Waksman Institute, Rutgers University, New Jersey, USA, 1:400), mouse Diap1 (gift from Bruce Hay, Division of Biology, California Institute of Technology, California, USA, 1:100), rabbit Vap33 (gift from Hugo Bellen, Department of Molecular and Human Genetics, Baylor College of Medicine, Houston, Texas, USA, 1:1000), rabbit aPKC (Santa Cruz Biotechnology, cat. no. sc216,  1:1000), rabbit Arf79F (gift from Maneesha S. Inamdar, Molecular Biology and Genetics Unit, Jawaharlal Nehru Centre for Advanced Scientific Research, Jakkur, Bangalore, India, 1:500; Khadilkar et al., 2014) and guinea pig Arf79F (gift from Fengwei Yu, Temasek Lifesciences Laboratory, Singapore, 1:200; Wang et al., 2017).

Secondary antibodies used were anti-mouse-IgG conjugated to Alexa Fluor 568, 633, 647, anti-rabbit-IgG conjugated to Alexa Fluor 568 and 633, and anti-guinea pig-IgG conjugated to Alexa 568 and 633 (all at 1:500). DNA was stained with 1 µM DAPI (stock prepared at 1 μg/ml, used at 1:1000; Sigma-Aldrich, cat. no. D9542).

### Affinity purification-mass spectrometry

Flies expressing endogenously YFP-tagged Vap33 (line #115288, Kyoto Stock Center) were grown in population cages, with embryos laid overnight collected on apple juice and agar plates. Extraction and protein purification were carried out as described previously (Neumuller et al., 2012). Briefly, extracts were prepared using Default Lysis Buffer (DLB) [50 mM Tris-HCl pH 7.5, 125 mM NaCl, 5% glycerol, 0.2% IGEPAL, 1.5 mM MgCl_2_, 1 mM DTT, 25 mM NaF, 1 mM Na_3_VO_4_, 1 mM EDTA, and 2× Complete protease inhibitor (Roche)], spun down to clarify, incubated first with empty agarose beads and then with GFP-Trap resin (ChromoTek), followed by washes and elution in SDS sample buffer. The eluates were loaded on SDS-polyacrylamide gels and electrophoresed so that the dye front migrated ∼1 cm in the separating gel. The gel was then stained with Coomassie Blue, and the lane was cut into two 5 mm×5 mm pieces and sent for mass spectrometry analysis (Taplin Mass Spectrometry Facility, Harvard Medical School, USA). Samples were digested with trypsin in-gel and peptides were analysed using a Thermo Scientific Orbitrap mass spectrometer. The mass spectrometry analysis was conducted on three biological replicates for both experimental samples (*Vap33-YFP*) and controls (*yw* fly line). Data were analysed using the Significance Analysis of Interactome (SAINT) program (Choi et al., 2011), and a complete table of Vap33–YFP-interacting proteins is included as Table S3. Mass spectrometry data were deposited to the ProteomeXchange Consortium via the PRIDE partner repository (Perez-Riverol et al., 2022) with the data set identifier PXD035110.

### co-IP and western blot analysis

Constructs used were: pMT-Vap33-V5 (Portela et al., 2018), pAc5.1-Flag-Hpo (Dent et al., 2015) and pAC5-1-HA-RtGEF (Dent et al., 2015). *Drosophila* S2 cells were maintained in standard Schneider's S2 medium with fetal bovine serum (Gibco) at 25°C, and transfections were performed using Effectene transfection reagent (Qiagen). CuSO_4_ was added to culture medium at a final concentration of 0.35 mM for inducing expression of Vap33–HA. Cells were lysed using DLB (see above) and spun down (21,000 ***g*** for 15 min) to remove debris. Clear cell lysates were incubated with anti-V5, anti-HA or anti-Flag beads (Sigma) for 2 h at 4°C. Beads were washed three times with lysis buffer, and protein complexes were eluted with SDS buffer. Proteins were separated via SDS-PAGE, blotted, incubated with primary and secondary antibodies, and signal was detected using the Odyssey imaging system (LI-COR). Primary antibodies used were: mouse anti-V5 (Sigma, cat. no. V8012, 1:1000), rabbit anti-Flag (Sigma, cat. no. F7425, 1:1000), rabbit anti-HA (Sigma, cat. no. H6908, 1:1000). Secondary antibodies used were: IRDye 800CW donkey anti-rabbit IgG (LI-COR), IRDye 680CW donkey anti-mouse IgG (LI-COR).

### Proximity ligation assay

The interactions between Lgl and aPKC, Vap33 or Git; Arf79F and Git, RtGEF or Hpo; and Vap33 and Git in *Drosophila* larval tissues were detected *in situ* using the Duolink^®^ In Situ Red Starter Kit Mouse/Rabbit (Sigma, DUO92101) according to the manufacturer's instructions. Briefly, primary antibody incubation was applied using the same conditions as immuno-histofluorescence staining. Duolink secondary antibodies against the primary antibodies were then added. These secondary antibodies were provided as conjugates to oligonucleotides that were able to form a closed circle through base pairing and ligation using Duolink ligation solution when the antibodies were in close proximity (Soderberg et al., 2006; a distance estimated to be <40 nm; Koos et al., 2014). The detection of the signals was conducted by rolling circle amplification using DNA polymerase incorporating fluorescently labelled nucleotides into the amplification products. The resulting positive signals were visualized as bright fluorescent dots, with each dot representing one interaction event. For a technical negative control, one of the primary antibodies was not added, with no positive signals were obtained from that assay. An additional negative control was performed in a tissue without one of the antigens (GFP) and the full protocol was performed in those tissues. As a positive control, antibodies against two well-known interactors in the tissues, Lgl–aPKC and Lgl–Vap33, were used. The tissues were visualized using confocal microscopy (Zeiss Confocal LSM 780 PicoQuant FLIM or Zeiss LSM 800 Airyscan laser scanning confocal).

Primary antibody pairs used were mouse GFP with rabbit Vap33 (Fig. 4A; Fig. S1G,H), mouse RFP with rabbit GFP (Fig. 4B; Fig. S1J), mouse RFP with rabbit Vap33 (Fig. 4C; Fig. S1K), mouse GFP with rabbit Arf79F (Fig. 4D; Fig. S1I), mouse RFP with rabbit Arf79F (Fig. 4E,F; Fig. S1L,M) and mouse GFP with rabbit aPKC (Fig. S1A,C). A GFP-tagged version of Lgl was used to detect interactions with Vap33, since the Lgl and Vap33 antibodies were both raised in rabbit. GFP-tagged Hpo was also used since the available Hpo antibody was raised in rats, and PLA antibodies designed for use with rat-raised antibodies were unavailable. RFP-tagged versions of RtGEF and Git were used for similar reasons.

### Imaging

Fluorescent-labelled samples were mounted in 80% glycerol or Vectashield (Vector Laboratories) and analysed by confocal microscopy (LEICA TCS SP5, Zeiss Confocal LSM 780 PicoQuant FLIM or Zeiss LSM 800 Airyscan laser scanning confocal). Images were processed using Leica LAS AF Lite and Fiji (Image J 1.50e). Images were assembled using Photoshop 21.2.3 (Adobe). Adult eyes were imaged on a dissecting microscope using a Scitec Infinity1 camera. Images were processed, analysed, and assembled using some combination of LAS AF Lite (Leica), Zen 2012 (Zeiss), Fiji and Photoshop 21.2.3 (Adobe).

### Statistical analysis of signal intensity

Relative *Ex-lacZ* (Fig. 1), or *E(spl)m8-lacZ* (Fig. S3) βGal stainings, and Diap1 stainings (Figs 2, 5, 6 and 7) within eye discs was determined using images taken at the same confocal settings. Average pixel intensity was measured using the measurement log tool from Fiji or Photoshop 5.1 (Adobe). In the case of *E(spl)m8-lacZ* analyses, clones were chosen just posterior to the morphogenetic furrow of each eye disc. Average pixel intensity was measured in mutant clones and the adjacent wild-type tissue of the same areas and expressed as a ratio of pixel intensity of the mutant clone relative to the wild-type tissue. To analyse and plot data, we used Microsoft Excel 2013 and GraphPad Prism 9. We performed a D'Agostino and Pearson normality test, and the data found to have a normal distribution were analysed by a two-tailed unpaired *t*-test with Welch correction. In the case of multiple comparisons, we used a one-way ANOVA with a Bonferroni post test. Error bars represent s.e.m.

## Supplementary Material



10.1242/joces.261917_sup1 Supplementary information

Table S3. Vap33 affinity purification-mass spectrometry interacting proteins.

## References

[JCS261917C1] Adarska, P., Wong-Dilworth, L. and Bottanelli, F. (2021). ARF GTPases and their ubiquitous role in intracellular trafficking beyond the golgi. *Front. Cell Dev. Biol.* 9, 679046. 10.3389/fcell.2021.679046 34368129 PMC8339471

[JCS261917C2] Archibald, A., Al-Masri, M., Liew-Spilger, A. and McCaffrey, L. (2015). Atypical protein kinase C induces cell transformation by disrupting Hippo/Yap signaling. *Mol. Biol. Cell* 26, 3578-3595. 10.1091/mbc.E15-05-0265 26269582 PMC4603929

[JCS261917C3] Asano, Y., Jimenez-Dalmaroni, A., Liverpool, T. B., Marchetti, M. C., Giomi, L., Kiger, A., Duke, T. and Baum, B. (2009). Pak3 inhibits local actin filament formation to regulate global cell polarity. *HFSP J.* 3, 194-203. 10.2976/1.3100548 19639041 PMC2714956

[JCS261917C4] Baek, S. H., Cho, H. W., Kwon, Y. C., Lee, J. H., Kim, M. J., Lee, H. and Choe, K. M. (2012). Requirement for Pak3 in Rac1-induced organization of actin and myosin during Drosophila larval wound healing. *FEBS Lett.* 586, 772-777. 10.1016/j.febslet.2012.01.061 22449966

[JCS261917C5] Bahri, S., Wang, S., Conder, R., Choy, J., Vlachos, S., Dong, K., Merino, C., Sigrist, S., Molnar, C., Yang, X. et al. (2010). The leading edge during dorsal closure as a model for epithelial plasticity: Pak is required for recruitment of the Scribble complex and septate junction formation. *Development* 137, 2023-2032. 10.1242/dev.045088 20501591

[JCS261917C6] Blanco, E., Ruiz-Romero, M., Beltran, S., Bosch, M., Punset, A., Serras, F. and Corominas, M. (2010). Gene expression following induction of regeneration in Drosophila wing imaginal discs. Expression profile of regenerating wing discs. *BMC Dev. Biol.* 10, 94. 10.1186/1471-213X-10-94 20813047 PMC2939566

[JCS261917C7] Borgese, N., Iacomino, N., Colombo, S. F. and Navone, F. (2021). The link between VAPB loss of function and amyotrophic lateral sclerosis. *Cells* 10, 1865. 10.3390/cells10081865 34440634 PMC8392409

[JCS261917C8] Bunker, B. D., Nellimoottil, T. T., Boileau, R. M., Classen, A. K. and Bilder, D. (2015). The transcriptional response to tumorigenic polarity loss in Drosophila. *Elife* 4, e03189. 10.7554/eLife.03189 25719210 PMC4369581

[JCS261917C9] Chen, L., Qin, F., Deng, X., Avruch, J. and Zhou, D. (2012). Hippo pathway in intestinal homeostasis and tumorigenesis. *Protein Cell* 3, 305-310. 10.1007/s13238-012-2913-9 22492181 PMC4875478

[JCS261917C10] Choi, H., Larsen, B., Lin, Z. Y., Breitkreutz, A., Mellacheruvu, D., Fermin, D., Qin, Z. S., Tyers, M., Gingras, A. C. and Nesvizhskii, A. I. (2011). SAINT: probabilistic scoring of affinity purification-mass spectrometry data. *Nat. Methods* 8, 70-73. 10.1038/nmeth.1541 21131968 PMC3064265

[JCS261917C11] Choi, S. W., Yeon, J. T., Park, K. I., Lee, C. H., Youn, B. S., Oh, J. and Lee, M. S. (2012). VapB as a regulator of osteoclastogenesis via modulation of PLCgamma2-Ca(2+)-NFAT signaling. *FEBS Lett.* 586, 263-269. 10.1016/j.febslet.2011.12.033 22245675

[JCS261917C12] Clark, B. S., Cui, S., Miesfeld, J. B., Klezovitch, O., Vasioukhin, V. and Link, B. A. (2012). Loss of Llgl1 in retinal neuroepithelia reveals links between apical domain size, Notch activity and neurogenesis. *Development* 139, 1599-1610. 10.1242/dev.078097 22492354 PMC3317966

[JCS261917C13] Collins, M. P. and Forgac, M. (2020). Regulation and function of V-ATPases in physiology and disease. *Biochimica. Et. Biophysica. Acta Biomembr.* 1862, 183341. 10.1016/j.bbamem.2020.183341 PMC7508768 32422136

[JCS261917C14] Conder, R., Yu, H., Zahedi, B. and Harden, N. (2007). The serine/threonine kinase dPak is required for polarized assembly of F-actin bundles and apical-basal polarity in the Drosophila follicular epithelium. *Dev. Biol.* 305, 470-482. 10.1016/j.ydbio.2007.02.034 17383630

[JCS261917C15] Deivasigamani, S., Verma, H. K., Ueda, R., Ratnaparkhi, A. and Ratnaparkhi, G. S. (2014). A genetic screen identifies Tor as an interactor of VAPB in a Drosophila model of amyotrophic lateral sclerosis. *Biol Open* 3, 1127-1138. 10.1242/bio.201410066 25361581 PMC4232771

[JCS261917C16] Dent, L. G., Poon, C. L., Zhang, X., Degoutin, J. L., Tipping, M., Veraksa, A. and Harvey, K. F. (2015). The GTPase regulatory proteins Pix and Git control tissue growth via the Hippo pathway. *Curr. Biol.* 25, 124-130. 10.1016/j.cub.2014.11.041 25484297 PMC5558436

[JCS261917C17] Dent, L. G., Manning, S. A., Kroeger, B., Williams, A. M., Saiful Hilmi, A. J., Crea, L., Kondo, S., Horne-Badovinac, S. and Harvey, K. F. (2019). The dPix-Git complex is essential to coordinate epithelial morphogenesis and regulate myosin during Drosophila egg chamber development. *PLoS Genet.* 15, e1008083. 10.1371/journal.pgen.1008083 31116733 PMC6555532

[JCS261917C18] Doggett, K., Grusche, F. A., Richardson, H. E. and Brumby, A. M. (2011). Loss of the Drosophila cell polarity regulator Scribbled promotes epithelial tissue overgrowth and cooperation with oncogenic Ras-Raf through impaired Hippo pathway signaling. *BMC Dev. Biol.* 11, 57. 10.1186/1471-213X-11-57 21955824 PMC3206446

[JCS261917C19] Dow, J. A. (1999). The multifunctional Drosophila melanogaster V-ATPase is encoded by a multigene family. *J. Bioenerg. Biomembr.* 31, 75-83. 10.1023/A:1005400731289 10340851

[JCS261917C20] Dow, J. A., Davies, S. A., Guo, Y., Graham, S., Finbow, M. E. and Kaiser, K. (1997). Molecular genetic analysis of V-ATPase function in Drosophila melanogaster. *J. Exp. Biol.* 200, 237-245. 10.1242/jeb.200.2.237 9050231

[JCS261917C21] Eaton, A. F., Merkulova, M. and Brown, D. (2021). The H+-ATPase (V-ATPase): from proton pump to signaling complex in health and disease. *Am. J. Physiol. Cell Physiol.* 320, C392-C414. 10.1152/ajpcell.00442.2020 33326313 PMC8294626

[JCS261917C22] Fahey-Lozano, N., La Marca, J. E., Portela, M. and Richardson, H. E. (2019). Drosophila models of cell polarity and cell competition in tumourigenesis. *Adv. Exp. Med. Biol.* 1167, 37-64. 10.1007/978-3-030-23629-8_3 31520348

[JCS261917C23] Felix, M., Chayengia, M., Ghosh, R., Sharma, A. and Prasad, M. (2015). Pak3 regulates apical-basal polarity in migrating border cells during Drosophila oogenesis. *Development* 142, 3692-3703. 10.1242/dev.125682 26395489

[JCS261917C24] Flaherty, M. S., Zavadil, J., Ekas, L. A. and Bach, E. A. (2009). Genome-wide expression profiling in the Drosophila eye reveals unexpected repression of notch signaling by the JAK/STAT pathway. *Dev. Dyn.* 238, 2235-2253. 10.1002/dvdy.21989 19504457 PMC2846647

[JCS261917C25] Frank, S. R. and Hansen, S. H. (2008). The PIX-GIT complex: a G protein signaling cassette in control of cell shape. *Semin. Cell Dev. Biol.* 19, 234-244. 10.1016/j.semcdb.2008.01.002 18299239 PMC2394276

[JCS261917C26] Gao, X. K., Sheng, Z. K., Lu, Y. H., Sun, Y. T., Rao, X. S., Shi, L. J., Cong, X. X., Chen, X., Wu, H. B., Huang, M. et al. (2023). VAPB-mediated ER-targeting stabilizes IRS-1 signalosomes to regulate insulin/IGF signaling. *Cell Discov.* 9, 83. 10.1038/s41421-023-00576-6 37528084 PMC10394085

[JCS261917C27] Grifoni, D., Garoia, F., Schimanski, C. C., Schmitz, G., Laurenti, E., Galle, P. R., Pession, A., Cavicchi, S. and Strand, D. (2004). The human protein Hugl-1 substitutes for Drosophila lethal giant larvae tumour suppressor function in vivo. *Oncogene* 23, 8688-8694. 10.1038/sj.onc.1208023 15467749

[JCS261917C28] Grifoni, D., Garoia, F., Bellosta, P., Parisi, F., De Biase, D., Collina, G., Strand, D., Cavicchi, S. and Pession, A. (2007). aPKCzeta cortical loading is associated with Lgl cytoplasmic release and tumor growth in Drosophila and human epithelia. *Oncogene* 26, 5960-5965. 10.1038/sj.onc.1210389 17369850

[JCS261917C29] Grifoni, D., Froldi, F. and Pession, A. (2013). Connecting epithelial polarity, proliferation and cancer in Drosophila: the many faces of lgl loss of function. *Int. J. Dev. Biol.* 57, 677-687. 10.1387/ijdb.130285dg 24395559

[JCS261917C30] Grusche, F. A., Richardson, H. E. and Harvey, K. F. (2010). Upstream regulation of the hippo size control pathway. *Curr. Biol.* 20, R574-R582. 10.1016/j.cub.2010.05.023 20619814

[JCS261917C31] Grusche, F. A., Degoutin, J. L., Richardson, H. E. and Harvey, K. F. (2011). The Salvador/Warts/Hippo pathway controls regenerative tissue growth in Drosophila melanogaster. *Dev. Biol.* 350, 255-266. 10.1016/j.ydbio.2010.11.020 21111727

[JCS261917C32] Grzeschik, N. A., Amin, N., Secombe, J., Brumby, A. M. and Richardson, H. E. (2007). Abnormalities in cell proliferation and apico-basal cell polarity are separable in Drosophila lgl mutant clones in the developing eye. *Dev. Biol.* 311, 106-123. 10.1016/j.ydbio.2007.08.025 17870065 PMC2974846

[JCS261917C33] Grzeschik, N. A., Parsons, L. M., Allott, M. L., Harvey, K. F. and Richardson, H. E. (2010). Lgl, aPKC, and Crumbs regulate the Salvador/Warts/Hippo pathway through two distinct mechanisms. *Curr. Biol.* 20, 573-581. 10.1016/j.cub.2010.01.055 20362447

[JCS261917C34] Guruharsha, K. G., Rual, J. F., Zhai, B., Mintseris, J., Vaidya, P., Vaidya, N., Beekman, C., Wong, C., Rhee, D. Y., Cenaj, O. et al. (2011). A protein complex network of Drosophila melanogaster. *Cell* 147, 690-703. 10.1016/j.cell.2011.08.047 22036573 PMC3319048

[JCS261917C35] Hanahan, D. and Weinberg, R. A. (2011). Hallmarks of cancer: the next generation. *Cell* 144, 646-674. 10.1016/j.cell.2011.02.013 21376230

[JCS261917C36] Harden, N., Lee, J., Loh, H. Y., Ong, Y. M., Tan, I., Leung, T., Manser, E. and Lim, L. (1996). A Drosophila homolog of the Rac- and Cdc42-activated serine/threonine kinase PAK is a potential focal adhesion and focal complex protein that colocalizes with dynamic actin structures. *Mol. Cell. Biol.* 16, 1896-1908. 10.1128/MCB.16.5.1896 8628256 PMC231177

[JCS261917C37] Harvey, K. and Tapon, N. (2007). The Salvador-Warts-Hippo pathway - an emerging tumour-suppressor network. *Nat. Rev. Cancer* 7, 182-191. 10.1038/nrc2070 17318211

[JCS261917C38] Huttlin, E. L., Ting, L., Bruckner, R. J., Gebreab, F., Gygi, M. P., Szpyt, J., Tam, S., Zarraga, G., Colby, G., Baltier, K. et al. (2015). The BioPlex network: a systematic exploration of the human interactome. *Cell* 162, 425-440. 10.1016/j.cell.2015.06.043 26186194 PMC4617211

[JCS261917C39] Imamura, N., Horikoshi, Y., Matsuzaki, T., Toriumi, K., Kitatani, K., Ogura, G., Masuda, R., Nakamura, N., Takekoshi, S. and Iwazaki, M. (2013). Localization of aPKC lambda/iota and its interacting protein, Lgl2, is significantly associated with lung adenocarcinoma progression. *Tokai J. Exp. Clin. Med.* 38, 146-158. 24318287

[JCS261917C40] Khadilkar, R. J., Rodrigues, D., Mote, R. D., Sinha, A. R., Kulkarni, V., Magadi, S. S. and Inamdar, M. S. (2014). ARF1-GTP regulates Asrij to provide endocytic control of Drosophila blood cell homeostasis. *Proc. Natl. Acad. Sci. USA* 111, 4898-4903. 10.1073/pnas.1303559111 24707047 PMC3977295

[JCS261917C41] Klezovitch, O., Fernandez, T. E., Tapscott, S. J. and Vasioukhin, V. (2004). Loss of cell polarity causes severe brain dysplasia in Lgl1 knockout mice. *Genes Dev.* 18, 559-571. 10.1101/gad.1178004 15037549 PMC374237

[JCS261917C42] Kobia, F., Duchi, S., Deflorian, G. and Vaccari, T. (2014). Pharmacologic inhibition of vacuolar H+ ATPase reduces physiologic and oncogenic Notch signaling. *Mol. Oncol.* 8, 207-220. 10.1016/j.molonc.2013.11.002 24309677 PMC5528540

[JCS261917C43] Koos, B., Andersson, L., Clausson, C. M., Grannas, K., Klaesson, A., Cane, G. and Soderberg, O. (2014). Analysis of protein interactions in situ by proximity ligation assays. *Curr. Top. Microbiol. Immunol.* 377, 111-126. 10.1007/82_2013_334 23921974

[JCS261917C44] Kuphal, S., Wallner, S., Schimanski, C. C., Bataille, F., Hofer, P., Strand, S., Strand, D. and Bosserhoff, A. K. (2006). Expression of Hugl-1 is strongly reduced in malignant melanoma. *Oncogene* 25, 103-110. 10.1038/sj.onc.1209008 16170365

[JCS261917C45] Kux, K. and Pitsouli, C. (2014). Tissue communication in regenerative inflammatory signaling: lessons from the fly gut. *Front. Cell Infect. Microbiol.* 4, 49. 10.3389/fcimb.2014.00049 24795868 PMC4006025

[JCS261917C46] Kwon, Y., Vinayagam, A., Sun, X., Dephoure, N., Gygi, S. P., Hong, P. and Perrimon, N. (2013). The Hippo signaling pathway interactome. *Science* 342, 737-740. 10.1126/science.1243971 24114784 PMC3951131

[JCS261917C47] La Marca, J. E. and Richardson, H. E. (2020). Two-faced: roles of JNK signalling during tumourigenesis in the drosophila model. *Front. Cell Dev. Biol.* 8, 42. 10.3389/fcell.2020.00042 32117973 PMC7012784

[JCS261917C48] Lee, T. and Luo, L. (2001). Mosaic analysis with a repressible cell marker (MARCM) for Drosophila neural development. *Trends Neurosci.* 24, 251-254. 10.1016/S0166-2236(00)01791-4 11311363

[JCS261917C49] Lim, D. H., Lee, S., Han, J. Y., Choi, M. S., Hong, J. S. and Lee, Y. S. (2019). MicroRNA miR-252 targets mbt to control the developmental growth of Drosophila. *Insect Mol. Biol.* 28, 444-454. 10.1111/imb.12562 30582233

[JCS261917C50] Lisovsky, M., Dresser, K., Baker, S., Fisher, A., Woda, B., Banner, B. and Lauwers, G. Y. (2009). Cell polarity protein Lgl2 is lost or aberrantly localized in gastric dysplasia and adenocarcinoma: an immunohistochemical study. *Mod. Pathol.* 22, 977-984. 10.1038/modpathol.2009.68 19407852

[JCS261917C51] Lisovsky, M., Dresser, K., Woda, B. and Mino-Kenudson, M. (2010). Immunohistochemistry for cell polarity protein Lgl2 differentiates pancreatic intraepithelial neoplasia-3 and ductal adenocarcinoma of the pancreas from lower-grade pancreatic intraepithelial neoplasias. *Hum. Pathol.* 41, 902-909. 10.1016/j.humpath.2009.12.004 20233622

[JCS261917C52] Lu, X., Feng, X., Man, X., Yang, G., Tang, L., Du, D., Zhang, F., Yuan, H., Huang, Q., Zhang, Z. et al. (2009). Aberrant splicing of Hugl-1 is associated with hepatocellular carcinoma progression. *Clin. Cancer Res.* 15, 3287-3296. 10.1158/1078-0432.CCR-08-2078 19447873

[JCS261917C53] Mao, D., Lin, G., Tepe, B., Zuo, Z., Tan, K. L., Senturk, M., Zhang, S., Arenkiel, B. R., Sardiello, M. and Bellen, H. J. (2019). VAMP associated proteins are required for autophagic and lysosomal degradation by promoting a PtdIns4P-mediated endosomal pathway. *Autophagy* 15, 1214-1233. 10.1080/15548627.2019.1580103 30741620 PMC6613884

[JCS261917C54] Melzer, J., Kraft, K. F., Urbach, R. and Raabe, T. (2013). The p21-activated kinase Mbt is a component of the apical protein complex in central brain neuroblasts and controls cell proliferation. *Development* 140, 1871-1881. 10.1242/dev.088435 23571212

[JCS261917C55] Menzel, N., Schneeberger, D. and Raabe, T. (2007). The Drosophila p21 activated kinase Mbt regulates the actin cytoskeleton and adherens junctions to control photoreceptor cell morphogenesis. *Mech. Dev.* 124, 78-90. 10.1016/j.mod.2006.09.007 17097274

[JCS261917C56] Menzel, N., Melzer, J., Waschke, J., Lenz, C., Wecklein, H., Lochnit, G., Drenckhahn, D. and Raabe, T. (2008). The Drosophila p21-activated kinase Mbt modulates DE-cadherin-mediated cell adhesion by phosphorylation of Armadillo. *Biochem. J.* 416, 231-241. 10.1042/BJ20080465 18636970

[JCS261917C57] Muthuswamy, S. K. and Xue, B. (2012). Cell polarity as a regulator of cancer cell behavior plasticity. *Annu. Rev. Cell Dev. Biol.* 28, 599-625. 10.1146/annurev-cellbio-092910-154244 22881459 PMC3997262

[JCS261917C58] Neumuller, R. A., Wirtz-Peitz, F., Lee, S., Kwon, Y., Buckner, M., Hoskins, R. A., Venken, K. J., Bellen, H. J., Mohr, S. E. and Perrimon, N. (2012). Stringent analysis of gene function and protein-protein interactions using fluorescently tagged genes. *Genetics* 190, 931-940. 10.1534/genetics.111.136465 22174071 PMC3296255

[JCS261917C59] Ntziachristos, P., Lim, J. S., Sage, J. and Aifantis, I. (2014). From fly wings to targeted cancer therapies: a centennial for notch signaling. *Cancer Cell* 25, 318-334. 10.1016/j.ccr.2014.02.018 24651013 PMC4040351

[JCS261917C60] Pamarthy, S., Kulshrestha, A., Katara, G. K. and Beaman, K. D. (2018). The curious case of vacuolar ATPase: regulation of signaling pathways. *Mol. Cancer* 17, 41. 10.1186/s12943-018-0811-3 29448933 PMC5815226

[JCS261917C61] Parsons, L. M., Grzeschik, N. A. and Richardson, H. E. (2014a). lgl regulates the hippo pathway independently of Fat/Dachs, Kibra/Expanded/Merlin and dRASSF/dSTRIPAK. *Cancers (Basel)* 6, 879-896. 10.3390/cancers6020879 24743776 PMC4074808

[JCS261917C62] Parsons, L. M., Portela, M., Grzeschik, N. A. and Richardson, H. E. (2014b). Lgl regulates Notch signaling via endocytosis, independently of the apical aPKC-Par6-Baz polarity complex. *Curr. Biol.* 24, 2073-2084. 10.1016/j.cub.2014.07.075 25220057

[JCS261917C63] Perez-Riverol, Y., Bai, J., Bandla, C., Garcia-Seisdedos, D., Hewapathirana, S., Kamatchinathan, S., Kundu, D. J., Prakash, A., Frericks-Zipper, A., Eisenacher, M. et al. (2022). The PRIDE database resources in 2022: a hub for mass spectrometry-based proteomics evidences. *Nucleic Acids Res.* 50, D543-D552. 10.1093/nar/gkab1038 34723319 PMC8728295

[JCS261917C64] Petzoldt, A. G., Gleixner, E. M., Fumagalli, A., Vaccari, T. and Simons, M. (2013). Elevated expression of the V-ATPase C subunit triggers JNK-dependent cell invasion and overgrowth in a Drosophila epithelium. *Dis. Model. Mech.* 6, 689-700. 10.1242/dmm.010660 23335205 PMC3634652

[JCS261917C65] Pojer, J. M., Manning, S. A., Kroeger, B., Kondo, S. and Harvey, K. F. (2021). The Hippo pathway uses different machinery to control cell fate and organ size. *iScience* 24, 102830. 10.1016/j.isci.2021.102830 34355153 PMC8322298

[JCS261917C66] Portela, M., Parsons, L. M., Grzeschik, N. A. and Richardson, H. E. (2015). Regulation of Notch signaling and endocytosis by the Lgl neoplastic tumor suppressor. *Cell Cycle* 14, 1496-1506. 25789785 10.1080/15384101.2015.1026515 PMC4613855

[JCS261917C67] Portela, M., Yang, L., Paul, S., Li, X., Veraksa, A., Parsons, L. M. and Richardson, H. E. (2018). Lgl reduces endosomal vesicle acidification and Notch signaling by promoting the interaction between Vap33 and the V-ATPase complex. *Sci. Signal.* 11, eaar1976. 10.1126/scisignal.aar1976 29871910 PMC6437781

[JCS261917C68] Recasens-Alvarez, C., Ferreira, A. and Milan, M. (2017). JAK/STAT controls organ size and fate specification by regulating morphogen production and signalling. *Nat. Commun.* 8, 13815. 10.1038/ncomms13815 28045022 PMC5216089

[JCS261917C69] Richardson, H. E. and Portela, M. (2017). Tissue growth and tumorigenesis in Drosophila: cell polarity and the Hippo pathway. *Curr. Opin. Cell Biol.* 48, 1-9. 10.1016/j.ceb.2017.03.006 28364663

[JCS261917C70] Sanhueza, M., Chai, A., Smith, C., McCray, B. A., Simpson, T. I., Taylor, J. P. and Pennetta, G. (2015). Network analyses reveal novel aspects of ALS pathogenesis. *PLoS Genet.* 11, e1005107. 10.1371/journal.pgen.1005107 25826266 PMC4380362

[JCS261917C71] Schimanski, C. C., Schmitz, G., Kashyap, A., Bosserhoff, A. K., Bataille, F., Schafer, S. C., Lehr, H. A., Berger, M. R., Galle, P. R., Strand, S. et al. (2005). Reduced expression of Hugl-1, the human homologue of Drosophila tumour suppressor gene lgl, contributes to progression of colorectal cancer. *Oncogene* 24, 3100-3109. 10.1038/sj.onc.1208520 15735678

[JCS261917C72] Schneeberger, D. and Raabe, T. (2003). Mbt, a Drosophila PAK protein, combines with Cdc42 to regulate photoreceptor cell morphogenesis. *Development* 130, 427-437. 10.1242/dev.00248 12490550

[JCS261917C73] Schroeder, M. C., Chen, C. L., Gajewski, K. and Halder, G. (2013). A non-cell-autonomous tumor suppressor role for Stat in eliminating oncogenic scribble cells. *Oncogene* 32, 4471-4479. 10.1038/onc.2012.476 23108407

[JCS261917C74] Soderberg, O., Gullberg, M., Jarvius, M., Ridderstrale, K., Leuchowius, K. J., Jarvius, J., Wester, K., Hydbring, P., Bahram, F., Larsson, L. G. et al. (2006). Direct observation of individual endogenous protein complexes in situ by proximity ligation. *Nat. Methods* 3, 995-1000. 10.1038/nmeth947 17072308

[JCS261917C75] Stephens, R., Lim, K., Portela, M., Kvansakul, M., Humbert, P. O. and Richardson, H. E. (2018). The scribble cell polarity module in the regulation of cell signaling in tissue development and tumorigenesis. *J. Mol. Biol.* 430, 3585-3612. 10.1016/j.jmb.2018.01.011 29409995

[JCS261917C76] Sun, S., Reddy, B. V. and Irvine, K. D. (2015). Localization of Hippo signalling complexes and Warts activation in vivo. *Nat. Commun.* 6, 8402. 10.1038/ncomms9402 26420589 PMC4598633

[JCS261917C77] Tsuda, H., Han, S. M., Yang, Y., Tong, C., Lin, Y. Q., Mohan, K., Haueter, C., Zoghbi, A., Harati, Y., Kwan, J. et al. (2008). The amyotrophic lateral sclerosis 8 protein VAPB is cleaved, secreted, and acts as a ligand for Eph receptors. *Cell* 133, 963-977. 10.1016/j.cell.2008.04.039 18555774 PMC2494862

[JCS261917C78] Vaccari, T., Duchi, S., Cortese, K., Tacchetti, C. and Bilder, D. (2010). The vacuolar ATPase is required for physiological as well as pathological activation of the Notch receptor. *Development* 137, 1825-1832. 10.1242/dev.045484 20460366 PMC2867318

[JCS261917C79] Venken, K. J., Schulze, K. L., Haelterman, N. A., Pan, H., He, Y., Evans-Holm, M., Carlson, J. W., Levis, R. W., Spradling, A. C., Hoskins, R. A. et al. (2011). MiMIC: a highly versatile transposon insertion resource for engineering Drosophila melanogaster genes. *Nat. Methods* 8, 737-743. 10.1038/nmeth.1662 21985007 PMC3191940

[JCS261917C80] Verghese, S. and Moberg, K. (2019). Roles of membrane and vesicular traffic in regulation of the hippo pathway. *Front. Cell Dev. Biol.* 7, 384. 10.3389/fcell.2019.00384 32010696 PMC6971369

[JCS261917C81] Vlachos, S. and Harden, N. (2011). Genetic evidence for antagonism between Pak protein kinase and Rho1 small GTPase signaling in regulation of the actin cytoskeleton during Drosophila oogenesis. *Genetics* 187, 501-512. 10.1534/genetics.110.120998 21098722 PMC3030492

[JCS261917C82] Wang, Y., Zhang, H., Shi, M., Liou, Y. C., Lu, L. and Yu, F. (2017). Sec71 functions as a GEF for the small GTPase Arf1 to govern dendrite pruning of Drosophila sensory neurons. *Development* 144, 1851-1862. 10.1242/dev.146175 28420712

[JCS261917C83] Zhou, W., Li, X. and Premont, R. T. (2016). Expanding functions of GIT Arf GTPase-activating proteins, PIX Rho guanine nucleotide exchange factors and GIT-PIX complexes. *J. Cell Sci.* 129, 1963-1974. 10.1242/jcs.179465 27182061 PMC6518221

