# Supplementary material for: The Drosophila tumour suppressor Lgl and Vap33 activate the Hippo pathway through a dual mechanism
Source: J Cell Sci. 2024 Feb 16;137(4):jcs261917. doi: 10.1242/jcs.261917 (PMC10911279; doi:10.1242/jcs.261917)
Supplement: Supplementary information [file joces-137-261917-s1.pdf]

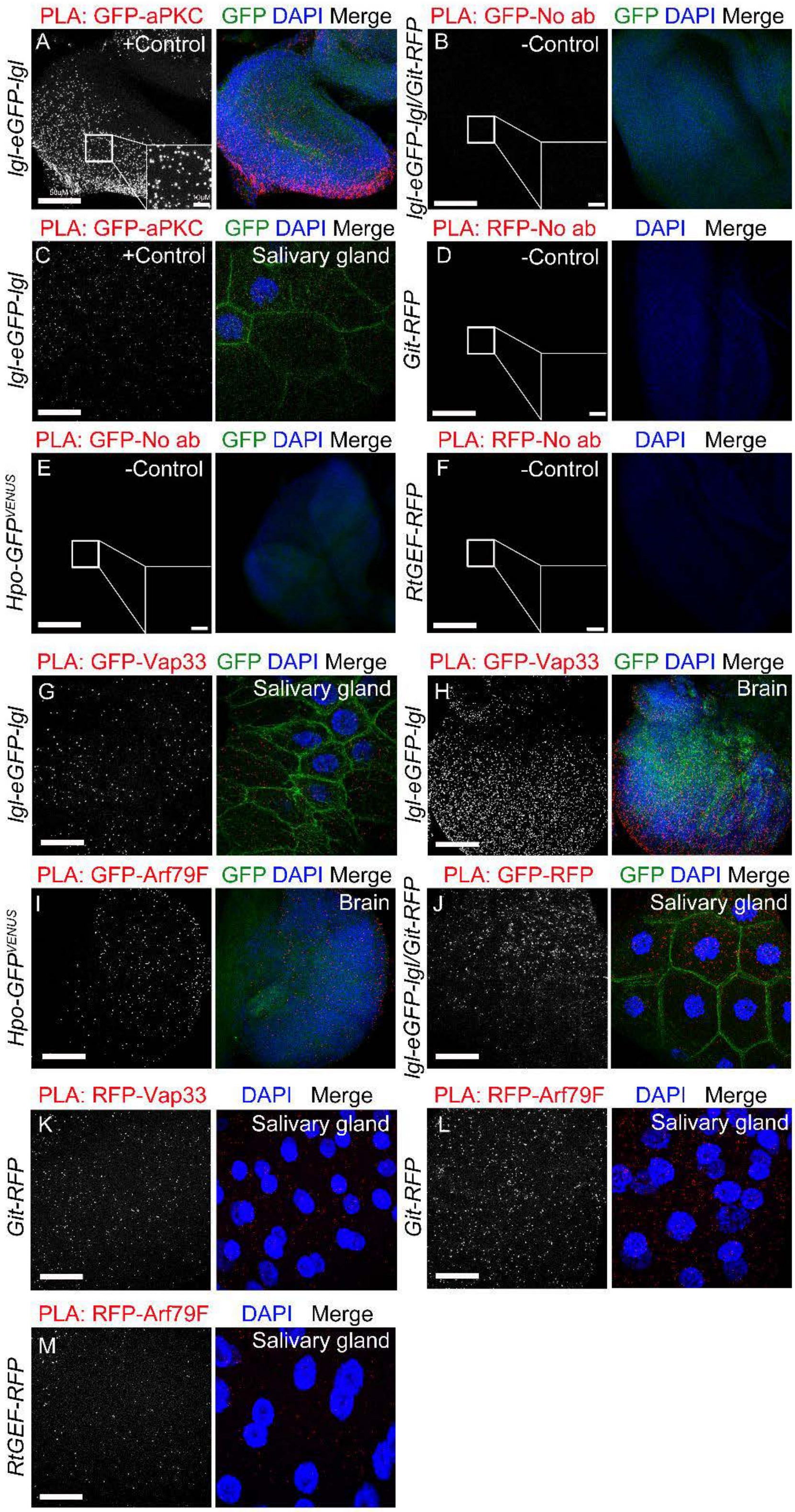

Journal of Cell Science • Supplementary information

Fig. S1. RtGEF interacts with Arf79F, Vap33, Lgl and Arf79F interact with Git in the salivary glands, and Arf79F in the brain.

(A-M) Confocal planar images showing *in situ* proximity ligation assay (PLA) on third instar larval eye discs, brains or salivary glands. Positive PLA results appear as punctate signals (grey, or red in the merges). Nuclei are stained with DAPI (blue). Insets show high magnification images of the PLA foci. (A) Positive-control PLA on *lgl-eGFP-lgl* eye discs using antibodies against GFP and aPKC. (B) Negative control PLA in *lgl-eGFP-lgl*/*Git-RFP* eye discs using only one primary antibody against GFP. (C) Positive-control PLA in *lgl-eGFP-lgl* salivary glands using antibodies against GFP and aPKC. (D) Negative control PLA on *Git-RFP* eye discs using only one primary antibody against RFP. (E) Negative control PLA in *Hpo-GFP* eye discs using only one primary antibody against GFP. (F) Negative control PLA in *RtGEF-RFP* eye discs using only one primary antibody against RFP. (G, H) Positive-control PLA on *lgl-eGFP-lgl* salivary glands (G) and brains (H) using antibodies against GFP and Vap33. (I) PLA in *Hpo-GFP* brains using antibodies against GFP and Arf79F. (J) PLA in *lgl-eGFP-lgl*/*Git-RFP* salivary glands using antibodies against RFP and GFP. (K) PLA in *Git-RFP* salivary glands using antibodies against RFP and Vap33. (L) PLA in *Git-RFP* salivary glands using antibodies against RFP and Arf79F. (M) PLA in *RtGEF-RFP* salivary glands using antibodies against RFP and Arf79F. Scale bars represent 50 µm.

Journal of Cell Science • Supplementary information

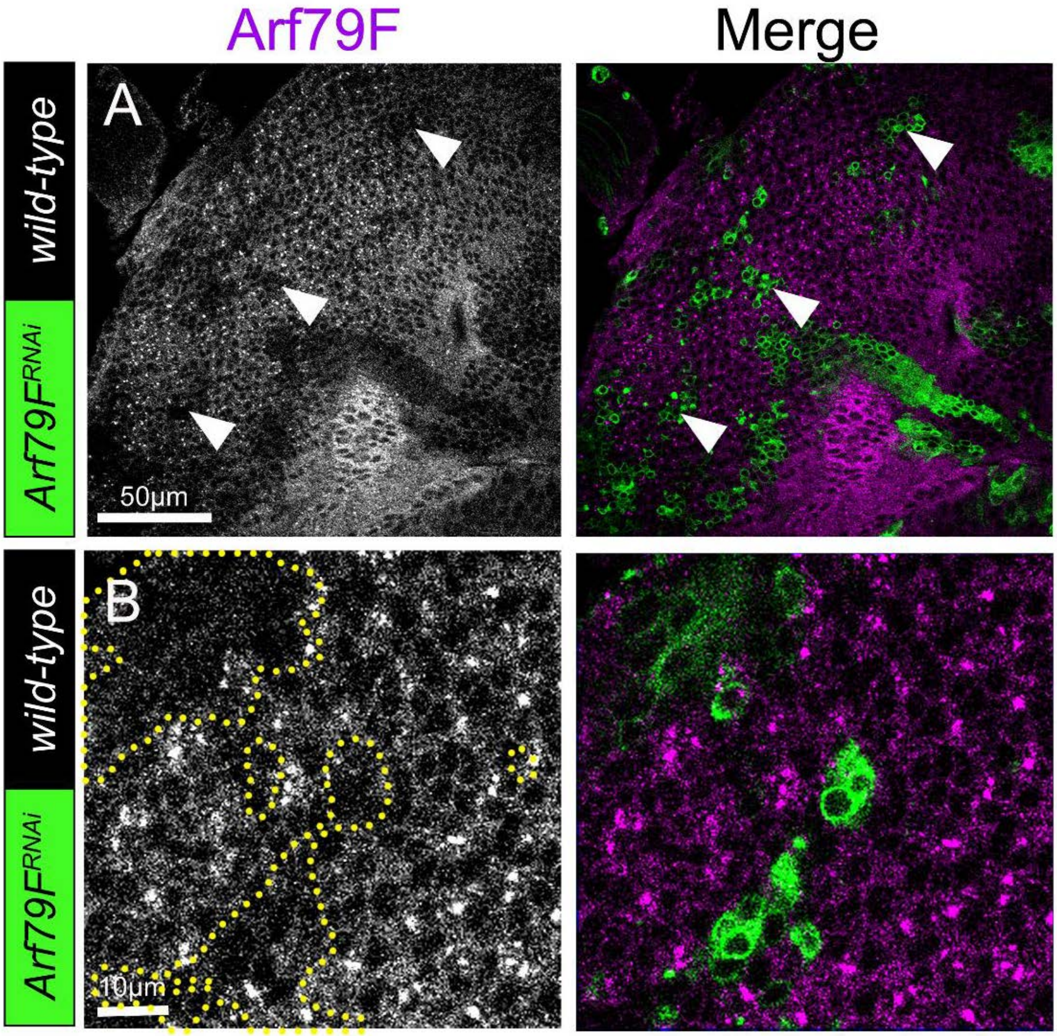

Fig. S2. Arf79F staining in *Arf79F* knockdown mosaic eye-antennal discs.

(A) Confocal planar sections of *Arf79F*^{RNAi} mosaic third instar larval eye-antennal discs (clones marked by GFP) stained for Arf79F (magenta). Knockdown of Arf79F using the *Arf79F*^{RNAi} line reduced the amount of Arf79F protein in the *Arf79F*^{RNAi} GFP-positive clones (green, example clones marked by arrowheads). Higher magnification shown in (B). Scale bars represent 50 µm (A) or 10 µm (B).

Journal of Cell Science • Supplementary information

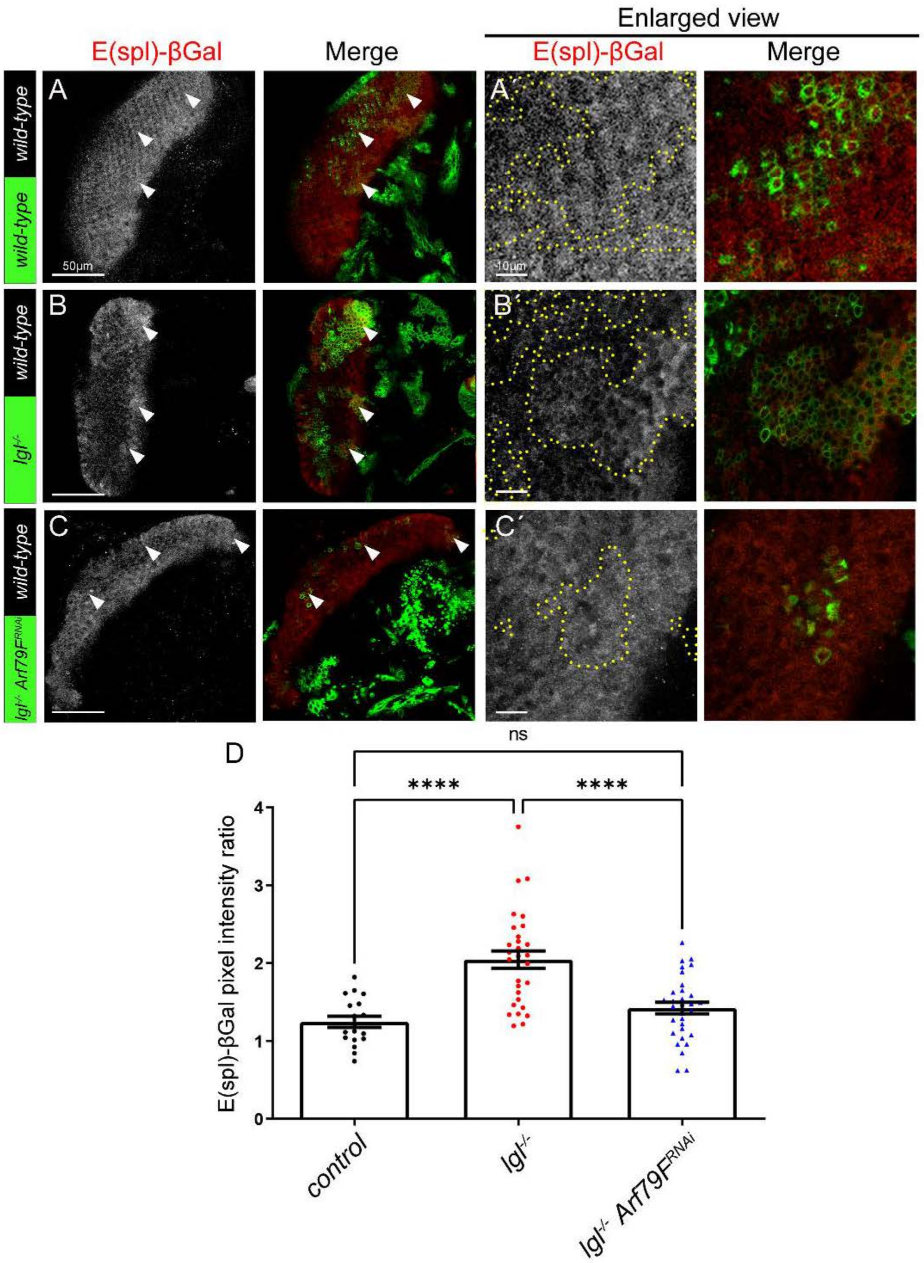

Fig. S3. Knockdown of *Arf79F* prevents Notch signalling pathway upregulation in *lgl* mutant tissue.

(A-C) Confocal planar sections of a mosaic eye discs containing the Notch target *E(spl)lacZ* reporter, stained for βGal (grey, or red in merges, example clones marked by arrowheads). (A) Control mosaic disc showing endogenous expression of *E(spl)lacZ*

Journal of Cell Science • Supplementary information

within and posterior to the morphogenetic furrow (arrowheads indicate clones showing normal *E(spl)lacZ* expression). (B) *lgl⁻* mosaic disc (mutant clones are GFP-positive). (C) *lgl⁻ Arf79F*^{RNAi} (mutant clones are GFP-positive). (D) Quantification of βGal pixel intensity ratio between *wild-type* clones compared to mutant/transgenic clones. Error bars represent SEM. **** P-value<0.0001 (one-way ANOVA with Bonferroni post-test). In all images, posterior is to the left, and the scale bars represent 50 µm. (A´, B´, C´) Higher magnifications of βGal stainings (grey, or red in merges, mutant clones are GFP-positive) for all genotypes. Scale bars represent 10 µm.

Journal of Cell Science • Supplementary information

Table S1. *Drosophila* stocks

***Drosophila* stock**	**Source and reference**
l(2)gl^{27S3} (denoted as *lgl*^{27S3})	(Grzeschik et al., 2007)
P(E(spl)m8-HLH-2.61)2 (denoted as *E(spl)lacZ*$^{m8-2.61}$ or *E(spl)lacZ,*)	A. Bergmann (Kramatschek and Campos-Ortega, 1994) on chromosome *2R* (Christiansen et al., 2013)
P(UAS-lacZ.nls) (denoted as *UAS-lacZ-nls (III))*	G. Baeg
P(UAS-Vha44.P) (denoted as *UAS-Vha44*)	M. Simons (Petzoldt et al., 2013)
Vha68-2^{R6} (EMS generated null mutant, due to stop codon mutation)	BL39621, Bloomington Stock Center (Vaccari et al., 2010)
P(y[+t7.7] w[+mC]=10XUAS-mCD8::GFP)attP2 (denoted as *UAS-CD8-GFP,*)	BL32184, Bloomington Stock Center
Mi(y[+mDint2]=MIC)l(2)gl[MI07575] (denoted as *MI07575 lgl-eGFP-lgl*	BL43734, Bloomington Stock Center (Venken et al., 2011)
P(w[+mC]=UAS-Vap-33-1.P)3 (denoted as *UAS-Vap33*)	BL26693, Bloomington Stock Center (Pennetta et al., 2002)
P(TRiP.JF01355)attP2 (denoted as *UAS-luciferase*^{RNAi})	BL31603, Bloomington Stock Center
P(ey3.5-FLP.B)1, y^{1} *w***; P(w(+mC)=UAS-mCD8::GFP.L)Ptp4E(LL4); P(w(+mC)=tubP-GAL80)LL10 P(ry(+t7.2)=neoFRT)40A; P(w(+mC)=tubP-GAL4)LL7*<br><br>(denoted as *ey-FLP, UAS-GFP; Tub-GAL80, FRT40A; Tub-GAL4/TM6B* (*ey-FLP MARCM 2L*)).	(Grzeschik et al., 2010)
y[1] w[*]; Pvr[1] P(ry[+t7.2]=neoFRT)40A (denoted as *FRT40A*).	BL58427, Bloomington Stock Center
w; Vha68-2 RNAi TRiP.HMS01056}attP2	BL34582, Bloomington Stock Center
w;; UAS-Arf79F^{RNAi}	VDRC 23082 and BL66175
w; UAS-Sec71^{DN}	Fengwei Yu (Wang et al., 2017)

w; Git-tRFP	(Dent et al., 2019)
w; Hpo-VENUS	(Pojer et al., 2021)
w; RtGEF-tRFP	(Dent et al., 2019)
w; FRT40A, $RtGEF^{1036}$/CyO-GFP	(Dent et al., 2019)
y v;; UAS-Git^{RNAi}	BL31583
w;; UAS-wts^{RNAi}	NIG 12072R-1
ex-lacZ	BL44248
yw hs-FLP;; Act>CD2>GAL4, UAS-GFP	R. Mann

Journal of Cell Science • Supplementary information

Table S2. List of genotypes of the samples used in each Figure.

**Figure 1**	**Genotype**
(A)	*yw hs-FLP; ex-lacZ/ CyO; Act>CD2>GAL4, UAS-GFP/UAS-luciferase*^{RNAi}
(B)	*yw hs-FLP; ex-lacZ/ CyO; Act>CD2>GAL4, UAS-GFP/UAS-Vha86-2*^{RNAi}
(C)	*yw hs-FLP; ex-lacZ/ CyO; Act>CD2>GAL4, UAS-GFP/UAS-wts*^{RNAi}
(D)	*yw hs-FLP; ex-lacZ/ CyO; Act>CD2>GAL4, UAS-GFP/UAS-Vha44*
**Figure 2**	
(A-B)	*w, eyFLP, UAS-GFP; lgl27S3, FRT40A; Tub-GAL80, FRT40A, Tub-GAL4*
(C-D)	*w, eyFLP, UAS-GFP; FRT40A; UAS-Vap33o/e /Tub-GAL80, FRT40A, Tub-GAL4*
(E-F)	*w, eyFLP, UAS-GFP; lgl27S3, FRT40A; UAS-Vap33o/e /Tub-GAL80, FRT40A, Tub-GAL4*
**Figure 4**	
(A)	*y,w; MiMIC lgl-eGFP-lgl MI07575*
(B)	*w; y,w; MiMIC lgl-eGFP-lgl MI07575/Git-RFP*
(C, E)	*w; Git-RFP*
(D)	*w; Hpo-GFP*
(F)	*w; RtGEF-RFP*
**Figure S1**	Related to Figure 4
(A, C, G, H)	*y,w; MiMIC lgl-eGFP-lgl MI07575*
(B, J)	*y,w; MiMIC lgl-eGFP-lgl MI07575/Git-RFP*
(D, K, L)	*w; Git-RFP*
(E, I)	*w; Hpo-GFP*
(F, M)	*w; RtGEF-RFP*
**Figure 5**	
(A-B)	*w, eyFLP, UAS-GFP; FRT40A; UAS-Vap33o/e /Tub-GAL80, FRT40A, Tub-GAL4*

(C-D)	*w, eyFLP, UAS-GFP; RtGEF*$^{-}$*, FRT40A; UAS-Vap33o/e /Tub-GAL80, FRT40A, Tub-GAL4*
**Figure 6**	
(A-B)	*w, eyFLP, UAS-GFP; FRT40A; Tub-GAL80, FRT40A, Tub-GAL4*
(C-D)	*w, eyFLP, UAS-GFP; Vha68-2*$^{-}$*, FRT40A; Tub-GAL80, FRT40A, Tub-GAL4*
(E-F)	*w, eyFLP, UAS-GFP; Vha68-2*$^{-}$*, FRT40A; UAS-Git*^{RNAi} */Tub-GAL80, FRT40A, Tub-GAL4*
**Figure 7**	
(A-B)	*w, eyFLP, UAS-GFP; FRT40A; Tub-GAL80, FRT40A, Tub-GAL4*
(C-D)	*w, eyFLP, UAS-GFP; lgl27S3, FRT40A; Tub-GAL80, FRT40A, Tub-GAL4*
(E-F)	*w, eyFLP, UAS-GFP; FRT40A; UAS-Arf79F*^{RNAi} */Tub-GAL80, FRT40A, Tub-GAL4*
(G-H)	*w, eyFLP, UAS-GFP; lgl27S3, FRT40A; UAS-Arf79F*^{RNAi} */Tub-GAL80, FRT40A, Tub-GAL4*
(I-J)	*w, eyFLP, UAS-GFP; FRT40A, UAS-Sec71*^{DN}*; Tub-GAL80, FRT40A, Tub-GAL4*
(K-L)	*w, eyFLP, UAS-GFP; lgl27S3, FRT40A, UAS-Sec71*^{DN}*; Tub-GAL80, FRT40A, Tub-GAL4*
**Figure S2**	Related to Figure 7
(A-B)	*w, eyFLP, UAS-GFP; FRT40A; UAS-Arf79F*^{RNAi} */Tub-GAL80, FRT40A, Tub-GAL4*
**Figure S3**	Related to Figure 7
(A)	*w, eyFLP, UAS-GFP; FRT40A, E(spl)m8-lacZ; Tub-GAL80, FRT40A, Tub-GAL4*
(B)	*w, eyFLP, UAS-GFP; lgl27S3, FRT40A, E(spl)m8-lacZ; Tub-GAL80, FRT40A, Tub-GAL4*
(C)	*w, eyFLP, UAS-GFP; lgl27S3, FRT40A, E(spl)m8-lacZ; UAS-Arf79F*^{RNAi} */Tub-GAL80, FRT40A, Tub-GAL4*

Table S3. Vap33 affinity purification-mass spectrometry interacting proteins.

Available for download at

https://journals.biologists.com/jcs/article-lookup/doi/10.1242/jcs.261917#supplementary-data

Journal of Cell Science • Supplementary information

Supplementary References

Christiansen, A. E., Ding, T., Fan, Y., Graves, H. K., Herz, H. M., Lindblad, J. L. and Bergmann, A. (2013). Non-cell autonomous control of apoptosis by ligand-independent Hedgehog signaling in Drosophila. *Cell Death Differ* **20**, 302-11.

Dent, L. G., Manning, S. A., Kroeger, B., Williams, A. M., Saiful Hilmi, A. J., Crea, L., Kondo, S., Horne-Badovinac, S. and Harvey, K. F. (2019). The dPix-Git complex is essential to coordinate epithelial morphogenesis and regulate myosin during Drosophila egg chamber development. *PLoS Genet* **15**, e1008083.

Grzeschik, N. A., Amin, N., Secombe, J., Brumby, A. M. and Richardson, H. E. (2007). Abnormalities in cell proliferation and apico-basal cell polarity are separable in Drosophila lgl mutant clones in the developing eye. *Dev Biol* **311**, 106-23.

Grzeschik, N. A., Parsons, L. M., Allott, M. L., Harvey, K. F. and Richardson, H. E. (2010). Lgl, aPKC, and Crumbs regulate the Salvador/Warts/Hippo pathway through two distinct mechanisms. *Curr Biol* **20**, 573-81.

Kramatschek, B. and Campos-Ortega, J. A. (1994). Neuroectodermal transcription of the Drosophila neurogenic genes E(spl) and HLH-m5 is regulated by proneural genes. *Development* **120**, 815-26.

Pennetta, G., Hiesinger, P. R., Fabian-Fine, R., Meinertzhagen, I. A. and Bellen, H. J. (2002). Drosophila VAP-33A directs bouton formation at neuromuscular junctions in a dosage-dependent manner. *Neuron* **35**, 291-306.

Petzoldt, A. G., Gleixner, E. M., Fumagalli, A., Vaccari, T. and Simons, M. (2013). Elevated expression of the V-ATPase C subunit triggers JNK-dependent cell invasion and overgrowth in a Drosophila epithelium. *Dis Model Mech* **6**, 689-700.

Pojer, J. M., Manning, S. A., Kroeger, B., Kondo, S. and Harvey, K. F. (2021). The Hippo pathway uses different machinery to control cell fate and organ size. *iScience* **24**, 102830.

Vaccari, T., Duchi, S., Cortese, K., Tacchetti, C. and Bilder, D. (2010). The vacuolar ATPase is required for physiological as well as pathological activation of the Notch receptor. *Development* **137**, 1825-32.

Venken, K. J., Schulze, K. L., Haelterman, N. A., Pan, H., He, Y., Evans-Holm, M., Carlson, J. W., Levis, R. W., Spradling, A. C., Hoskins, R. A. et al. (2011). MiMIC: a highly versatile transposon insertion resource for engineering Drosophila melanogaster genes. *Nat Methods* **8**, 737-43.

Wang, Y., Zhang, H., Shi, M., Liou, Y. C., Lu, L. and Yu, F. (2017). Sec71 functions as a GEF for the small GTPase Arf1 to govern dendrite pruning of Drosophila sensory neurons. *Development* **144**, 1851-1862.
