# Supplementary material for: The Drosophila tumour suppressor Lgl and Vap33 activate the Hippo pathway through a dual mechanism
Source: J Cell Sci. 2024 Feb 16;137(4):jcs261917. doi: 10.1242/jcs.261917 (PMC10911279; doi:10.1242/jcs.261917)
Supplement: Supplementary file 1 [file joces-137-261917_review_history.pdf]

The *Drosophila* tumour suppressor Lgl and Vap33 activate the Hippo pathway through a dual mechanism

Marta Portela, Swastik Mukherjee, Sayantanee Paul, John E. La Marca, Linda M. Parsons, Alexey Veraksa, and Helena E. Richardson

DOI: 10.1242/jcs.261917

Editor:

Review timeline

Submission to Review Commons:	17 July 2023
Original submission:	19 December 2023
Accepted:	10 January 2024

Reviewer 1

Evidence, reproducibility and clarity

The manuscript entitled "The Drosophila Tumour Suppressor Lgl and Vap33 activate the Hippo pathway by a dual mechanism, involving RtGEF/Git/Arf79F and inhibition of the V- ATPase." by Portela et al. presents an interesting perspective of the molecular mechanism regulating Hippo pathway, revealing new proteins involved in this process.

In this study, the authors try to show us that Lgl activates the Hippo pathway via Vap33 either by interacting with RtGEF/Git/Arf79F or by inhibiting V-ATPase, thus controlling epithelial tissue growth.
The methodology used by the authors is adequate but could benefit from further experiments that would allow them to reach the conclusions stated in their research.

Thus, based on the interpretation of the results presented by the authors some concerns were raised that should be addressed during the review process and that are explained in the major comments.

Major comments:

- It is not clear why in "The Hippo signaling pathway is negatively regulated by V-ATPase activity in Drosophila" section, the authors use Vha68-2 RNAi to reduce the activity of V- ATPase and later they use the overexpression of Vha44 to activate V-ATPase. The authors should explain why they used different proteins to regulate V-ATPase. The way the authors wrote their results sounds like different Vha proteins regulate V-ATPase, which means that cells may have different ways to activate V-ATPases, not being clear if regardless that the downstream effect of V-ATPase activation is always reflected in the Hippo pathway. Thus, the authors should state what other Vha proteins may have a similar effect, I would like to see evidence that Vha44 and Vha68 knockdown and overexpression leads to similar results.
- In "Vap33 activates the Hippo pathway" section, the authors' conclusions represent a big statement considering the results obtained. Though Diap1 is a Hippo pathway target, it does not mean that this protein is solely regulated by this pathway. For example, there are studies that show that this gene can also be transcribed by STAT activity. Though in the following section the authors show how Vap33 activates this pathway, the results obtained in the section "Vap33 activates the Hippo pathway" are not enough to make this assumption. We suggest that the authors rephrase this section. (Optional: To maintain this statement, the authors should have performed,

© 2024. Published by The Company of Biologists under the terms of the Creative Commons Attribution License (https://creativecommons.org/licenses/by/4.0/).

for example, a luciferase assay containing specifically Hippo pathway binding sites in the Diap1 gene, showing that the transcription factor of the Hippo pathway is somehow regulated by Vap33).
- The authors present a highly speculative discussion, raising different hypotheses. Though such hypotheses are well supported by the literature, the authors would enrich the quality of their research if indeed they could prove them. Particularly, testing for vesicle acidification, testing if V-ATPase indeed blocks the interaction of Lgl/Vap33/RtGEF/Git/Arf79F, and alters Hpo localization, testing if Git/RtGEF inhibits Arf79F and consequent Hpo localization.
- The authors should also apply more specific techniques to infer how the Hippo pathway is affected by such genetic manipulation since diap1 can be a target gene of different pathways.

Minor comments:

- The authors present a well-structured manuscript, that generally is easy to understand. However, at some points, the statements given by the authors seem highly speculative.
- The figures presented in this manuscript and the statistical analysis seem adequate and are clearly described.

Significance

The study presented by Portela et al. gives new insights into the regulation of the Hippo pathway with the discovery of new proteins involved in this mechanism, which can be interesting to those working on basic research and focused on studying signal transduction. However, this study lacks some novelty.
Throughout the manuscript, the authors only observed the physiological consequences of manipulating this pathway based on the eye phenotypes, and in the discussion, many hypotheses were raised based on the already available literature, which shows that much is already known about the Hippo pathway.

The advances shown in this study are limited to the description of the signaling pathway itself and to the eye morphology. As a suggestion, the authors should explore the knowledge of their findings in order to understand how we can use them to achieve advances in other fields and physiological conditions. For example, only at the end of the discussion, did the authors raise the questions that would really push their discoveries a step forward, namely how this mechanism acts during the response to tissue wounding and whether the mammalian orthologs of Lgl and Vap33 also act via these mechanisms to control tissue growth in mammals. It would be interesting if the authors could direct their research efforts to understand if the proteins identified can be targeted to improve wound healing or to delay aging for example.

Altogether, the authors present an interesting study but, at this moment, it still lacks the significance and novelty needed for publication. We encourage the authors to keep up their good work to address these suggestions, which will definitely improve the quality of their study.

Reviewer 2

Evidence, reproducibility and clarity

Summary

This manuscript investigates potential mechanisms through which the lgl gene might affect the Hippo signaling pathway. The authors employ a combination of physical interaction studies and clonal analysis in Drosophila eye discs to investigate potential links between lgl and other genes. Some of the results are intriguing, but the analysis is rather preliminary, and there are technical concerns with some of the results presented.

Main issues

- The authors propose effects of genes involved in vesicle trafficking and acidification in Hippo signaling, but there is no clear cellular mechanism described by which these effects could be mediated. This deserves further consideration. eg if they think there are effects on the

© 2024. Published by The Company of Biologists under the terms of the Creative Commons Attribution License (https://creativecommons.org/licenses/by/4.0/).

localization of Hippo, this could be directly examined. In the Discussion, the authors suggest that "The V-ATPase might therefore act to inhibit Hippo pathway signalling by blocking the interaction of Lgl/Vap33/RtGEF/Git/Arf79F with Hpo in vesicles, thereby altering Hpo localization and inhibiting its activity." but Hippo is a cytoplasmic protein and has never been reported to be within vesicles.

- The Yki stains in Fig. 1 are confusing. The nature of the signal throughout the wing disc looks very different in 1A vs 1B vs 1C, this needs to be explained or re-examined. Fig 1C (wts RNAi) seems to show an elevated Yki signal in some cells, and lower in others in - prior studies have reported that wts affects the nuclear vs cytoplasmic localization of Yki, but not its levels, so this needs to be clarified.
- In Fig 1D the clones appear to have different effects in different regions of the eye disc; the authors should clarify. Also, the disc in 1D appears much younger than the discs in 1A-C, but similar age discs should be used for all comparisons.
- The authors should clarify whether any the manipulations they perform are associated with Jnk activation, as this could potentially provide an alternative explanation for downregulation of Hippo signaling.
- The authors report in Fig 2C,E that over-expression of Vap33 reduced expression of Diap1, which they interpret as evidence of increased Hippo pathway activity, but this experiment is lacking essential controls, as the apparent reduction of Diap1 could simply reflect increased cell death or a change in focal plane, and indeed the difference in the label stain makes it look like these cells are undergoing apoptosis. Thus it's important to also have a stain for a neutral protein, or at least a DNA stain. Additionally, it is important to stain for at least one additional marker of Hippo pathway activity (eg ex-lacZ or Yki localization), as there are other pathways that regulate Diap1
- In Fig. 4 the authors perform PLA experiments to examine potential association between various pairs of proteins, but they don't show us key controls. They report in the text using single antibodies as negative controls, but this doesn't control for non-specific localization of antibodies. The better negative control is to do the PLA experiments in parallel on tissues lacking the protein being detected (eg from animals not expressing the GFP- or RFP-tagged proteins they are examining). Also, there is a lot of variation in the apparent signals shown in different PLA experiments in fig 4, the authors should comment on this.
- The authors claim that RtGEF mutant cells increase Diap1 expression, and that Vap33 over-expression reverses this effect (Fig. 5). The effect of RtGEF looks very subtle and variable, it should be confirmed by examining additional reporters of Hippo pathway activity. It also seems like the disc in 5A is at a different stage &/or the quantitation is done from a different region as compared to the disc in 5C.
- The analysis of the influence of Vha68-2 mutant clones, and their genetic interaction with Git, similarly suffers from missing controls and incomplete analysis. Additional Hippo reporters besides just Diap1 should be examined. The Diap1 analysis which shows reduced expression needs examination of neutral controls or nuclear markers to assess potential apoptosis within clones, or changes in focal plane.

Similarly, the analysis of Arf79F mutant clones in Fig 7E,G is compromised by lack of controls for viability and tissue layer, and analysis of an additional Hippo reporter is once again essential.

Significance

The strength of the study is the potential dissection of novel connections between the lgl tumor suppressor and the Hippo pathway. However, there are signifiant limitations due to the preliminary nature of the study, which is incomplete and missing essential controls.
If these limitations are overcome the work will be of interest to specialists in the field.

Reviewer 3

Evidence, reproducibility and clarity

In this study, Portela and colleagues identified new regulators of Hippo pathway downstream of the core apico-basal polarity protein Lgl. While the impact of Lgl depletion of Yki activation was already characterised both in Drosophila and Vertebrates, the mechanism connecting these two pathways was still unclear. Using the Drosophila eye, mosaic analysis, epistatic analysis and

© 2024. Published by The Company of Biologists under the terms of the Creative Commons Attribution License (https://creativecommons.org/licenses/by/4.0/).

mass-spectrometry, they identified two routes through which Lgl depletion can lead to Hippo pathway downregulation and eye overgrowth. This regulation required the previously characterised Lgl interactor Vap33, which on the one hand activates Hippo by inhibiting the V-ATPase, and on the other hand activates Hippo through its interactions with the actin regulators Git, RtGEF (two previously characterised regulators of Hippo, https://pubmed.ncbi.nlm.nih.gov/25484297/) . They also identified another regulator of Hippo downstream of Lgl, Arf79C, whose ortholog interact with Git in mammals and is also in close proximity with Hippo, Git and RtGEF in Drosophila, and whose depletion abolish Hippo downregulation and eye overgrowth in Lgl mutant.

This is a well performed study which identified new links between Lgl and regulation of the Hippo pathway. Many of them are conserved in mammals and may be relevant in pathological conditions associated with Lgl loss of function and Yap missregulation. The experiments are well conducted with a quite thorough epistatic analysis combined with many assays to characterize protein interactions. Admittedly, the molecular mechanism remains uncharacterised and some experiments may help to indicate putative mechanisms, but the characterisation of these news regulators and clear genetic interactions results constitute already solid and interesting data. I have some suggestions though that could help to reinforce the conclusions.

Main suggestions:

1. While the precise molecular mechanisms is not absolutely necessary, it would be interesting to document the subcellular localisation of these new Hippo regulators in WT and Lgl mutant context (Git, RtGEF Vap33 and Arf79F), either with Antibody if they exist, or with fusion protein (which for a good part were already generated for the PLA results). This may reveal obvious misslocalisation which would support the role of Lgl as a scaffolding protein that maintain proper subcellular localisation of these factors.
2. Most of the epistatic experiments focus on factors that rescue the overgrowth and increase of diap1 expression in Lgl mutant. Did the author test if any of these core regulators are sufficient to recapitulate Lgl mutant eye phenotype, for instance Vap33 KD in the eye, or Ar79C overexpression. Negative results would still be informative as they would point to the existence of other downstream regulators of the eye phenotype
3. It is at the moment hard to interpret the relevance of the results obtained by PLA. While there are some negative controls based on the absence of secondary antibody, what is the number of particle obtained for two non-interacting cortical proteins ? Since this is based on proximity, I would expect that some positive particles would still appear by chance, but much less than for two physically interacting proteins or subunits of a complex. Could the author provide such a negative control by testing for instance Git/RtGEF with another non- interacting cortical protein ? That would help to assess the relevance of the conclusions based on PLA.
4. Some of the epistatic links are a bit hard to interpret at the moment, and additional epistatic test may be relevant. For instance, the increase of diap1 upon Git depletion in the Vha68 mutant (Figure 6) is used to conclude that Git is required for the Hippo upregulation upon reduced V-ATPase activity. However this could be compatible with two independent branches regulating Hippo (in an opposite manner), which is more less what is suggested by the authors in their conclusion and the model of figure 8. I would suggest to reformulate this conclusion in the result part. Similarly, there is currently no experimental exploration of the epistatic link between Arf68C, Git and RtGEF (which is based on results in mammals). It would be interesting to check if Git and RtGEF mutant phenotype (Hippo downregulation) can also be suppressed by downregulation of Arf79C.
5. Apart from very obvious phenotype (eye in Lgl mutant mosaic) it is a bit hard to interpret the picture of adult eye provided in this study (specially for mild phenotype). Could the authors provide more explanation in the legends , and if possible some quantitative evaluation of the phenotype when relevant ? Otherwhise, apart from obvious rescue of the Lgl mutant, it is a bit hard to interpret the other genotypes (e.g. : Vap33OE, RtGEF mutant, Vha68 mutant)

Other minor points:

1. I would recommend when possible to clearly indicate in Figure 8 which part of the pathway are clearly documented in this study, and which part are still hypothetical (eg: link with PAK).
2. Page 4, the sentence "as aPKC's association with the Hpo orthologs, MST1/2, and uncoupling

© 2024. Published by The Company of Biologists under the terms of the Creative Commons Attribution License (https://creativecommons.org/licenses/by/4.0/).

MST from the downstreamkinase, LATS (Wts), thereby leading to increased nuclear YAP (Yki) activity [17], consistent with what we observe in Drosophila [5]." may need to be reformulated (at least I had trouble to understand it).
3. Page 11 : "a decrease in Diap1 expression was observed and clones were smaller than wild-type clones (Fig 7E),suggesting that the Arf79F knockdown clones were being out- competed"
I am not sure one can conclude from this that the clone are "outcompeted" (which would suggest at context dependent disappearance of clone, while here the data could be totally compatible with a cell-autonomous decrease of growth and survival). This statement would only make sense if global eye depletion of Ar79F had no adult eye phenotype.

Significance

This study identifies regulators of Hippo which through their interactions with Vap33 explains for the first time how Lgl depletion leads to Hippo misregulation (without impairing apico- basal polarity). This is an interesting study based on epistatic analysis and mass- spectrometry and identify several regulators conserved in mammals. While the molecular mechanism remained to be explored, it clarifies for the first time how Lgl depletion (a core regulator of apico-basal polarity) leads to Hippo downregulation and tissue overgrowth, a phenotype also observed in mammals and characterised several years ago in Drosophila. The authors previously characterised the interaction between Vap33 and Lgl and its role in the regulation of Notch signaling through the V-ATPase. This study nicely complement these previous results and connect now Vap33 with Hippo and Lgl while answering a long unresolved question (how Lgl depletion affect Hippo pathway).

This results will be interesting for the large community studying the hippo pathway, apico- basal polarity and tissue growth. It also outlines interesting factors that could be relevant for tumour neoplasia and hyperplasia.

I have expertise in epithelial biology, apoptosis, cell competition, Drosophila, cell extrusion, mechanobiology, morphogenesis and growth regulation.

Author response to reviewers' comments

Manuscript number: RC-xx-xx
Corresponding author(s): First name, Last name

[Please use this template only if the submitted manuscript should be considered by the affiliate journal as a full revision in response to the points raised by the reviewers.

If you wish to submit a preliminary revision with a revision plan, please use our "Revision Plan" template. ***It is important to use the appropriate template to clearly inform the editors of your intentions.]***

1. **General Statements [optional]**

This section is optional. Insert here any general statements you wish to make about the goal of the study or about the reviews.

Unfortunately, my lab has now closed, and I was unable to get any additional information from the first author since she has moved back to Spain and left science, so we were unable to add any more data to address the reviewers' comments. However, we believe our arguments and edits to the text have covered the issues raised by all of the reviewers and present a substantially improved manuscript.

2. **Point-by-point description of the revisions**

© 2024. Published by The Company of Biologists under the terms of the Creative Commons Attribution License (https://creativecommons.org/licenses/by/4.0/).

This section is mandatory. Please insert a point-by-point reply describing the revisions that were already carried out and included in the transferred manuscript.

Reviewer #1 (Evidence, reproducibility and clarity (Required)):

The manuscript entitled "The Drosophila Tumour Suppressor Lgl and Vap33 activate the Hippo pathway by a dual mechanism, involving RtGEF/Git/Arf79F and inhibition of the V-ATPase." by Portela et al. presents an interesting perspective of the molecular mechanism regulating Hippo pathway, revealing new proteins involved in this process.
In this study, the authors try to show us that Lgl activates the Hippo pathway via Vap33 either by interacting with RtGEF/Git/Arf79F or by inhibiting V-ATPase, thus controlling epithelial tissue growth.
The methodology used by the authors is adequate but could benefit from further experiments that would allow them to reach the conclusions stated in their research.
Thus, based on the interpretation of the results presented by the authors some concerns were raised that should be addressed during the review process and that are explained in the major comments.

Major comments:

- It is not clear why in "The Hippo signaling pathway is negatively regulated by V-ATPase activity in Drosophila" section, the authors use Vha68-2 RNAi to reduce the activity of V-ATPase and later they use the overexpression of Vha44 to activate V-ATPase. The authors should explain why they used different proteins to regulate V-ATPase. The way the authors wrote their results sounds like different Vha proteins regulate V-ATPase, which means that cells may have different ways to activate V-ATPases, not being clear if regardless that the downstream effect of V-ATPase activation is always reflected in the Hippo pathway. Thus, the authors should state what other Vha proteins may have a similar effect, I would like to see evidence that Vha44 and Vha68 knockdown and overexpression leads to similar results.

Response: Vha68-2 and Vha44 are both components of the V-ATPase. We have added further details to the results to make this clearer. We have previously shown that knocking down several components of the V-ATPase, which disrupt V-ATPase function, have a similar effect on the Notch pathway (Portela et al., 2018 Sci. Signal., PMID: **29871910**). Vha44 overexpression had been documented to result in V-ATPase activation (Petzoldt et al., 2013, Dis Model Mech., PMID: **23335205**), and no other Drosophila V-ATPase transgenes were available to conduct experiments with other lines.

- In "Vap33 activates the Hippo pathway" section, the authors' conclusions represent a big statement considering the results obtained. Though Diap1 is a Hippo pathway target, it does not mean that this protein is solely regulated by this pathway. For example, there are studies that show that this gene can also be transcribed by STAT activity. Though in the following section the authors show how Vap33 activates this pathway, the results obtained in the section "Vap33 activates the Hippo pathway" are not enough to make this assumption. We suggest that the authors rephrase this section. (Optional: To maintain this statement, the authors should have performed, for example, a luciferase assay containing specifically Hippo pathway binding sites in the Diap1 gene, showing that the transcription factor of the Hippo pathway is somehow regulated by Vap33).

Response: Whilst Jak-STAT signalling has been shown to induce Diap1 expression in the wing disc during development (PMID: **28045022**), however expression profiling after activation of the Jak-STAT signalling in the eye epithelium did not identify Diap1 as a target (PMID: **19504457**). Additionally, there are no reports that Lgl depletion in eye disc clones elevates Jak-STAT signalling (Stephens et al., J. Mol. Biol. 2018, PMID: **29409995**), but instead loss of cell polarity in *scrib* mutant cells in the eye disc results in expression of the Jak-STAT pathway ligand, Upd, and non-cell autonomous induction of Jak-STAT signalling in the surrounding wild-type cells (PMID: **25719210**, PMID: **23108407**). We have previously shown that Lgl depletion leads to inactivation of the Hippo pathway and elevates expression of the canonical Yki targets, Ex and Diap1 (Grzeschik et al., 2010, Curr Biol., PMID: **20362447**). In this current study we show that Vap33 overexpression leads to the downregulation of Diap1 and in lgl mutant tissue reduces the elevated

© 2024. Published by The Company of Biologists under the terms of the Creative Commons Attribution License (https://creativecommons.org/licenses/by/4.0/).

Diap1 expression. Since there is no evidence that either Lgl or Vap33 (VAPB) perturbations affect the Jak-STAT signalling pathway, we conclude from our results that Vap33 acts by reducing Yki activity and thus activating the Hippo pathway. We have added additional explanation to this section of our manuscript.

- The authors present a highly speculative discussion, raising different hypotheses. Though such hypotheses are well supported by the literature, the authors would enrich the quality of their research if indeed they could prove them. Particularly, testing for vesicle acidification, testing if V-ATPase indeed blocks the interaction of Lgl/Vap33/RtGEF/Git/Arf79F, and alters Hpo localization, testing if Git/RtGEF inhibits Arf79F and consequent Hpo localization.

Response: Although it would extend the paper to conduct further experiments, my lab is now closed so this is not possible. We have already published that vesicle acidification is increased in lgl mutant tissue (Portela et al., 2018, Sci. Signal., PMID: **29871910**) and that Hpo localization is altered in lgl mutant tissue (Grzeschik et al., 2010, Curr. Biol., PMID: **20362447**).

- The authors should also apply more specific techniques to infer how the Hippo pathway is affected by such genetic manipulation since diap1 can be a target gene of different pathways.

Response: We have shown that lgl mutant tissue also shows upregulation of the Hippo pathway target, Ex-LacZ, and affects the phosphorylation of Yki (Grzeschik et al., 2010, Curr. Biol., PMID: **20362447**), and RtGEF/Git mutant tissue shows upregulation of the Yki target, Ex-LacZ (Dent et al., 2015, Curr. Biol., PMID: **25484297**). Since RtGEF/Git are positive regulators of Hippo, but there is no evidence that they are involved in the regulation of the Jak-STAT pathway, the effect of Vap33 overexpression on Diap1 levels in the context of a RtGEF knockdown (Fig 5) is most likely to be due to effects on the Hippo pathway. Similarly, since Lgl deficiency upregulates Yki targets, Ex-LacZ and Diap1 (Grzeschik et al., 2010, Curr. Biol., PMID: **20362447**), the reduction of the elevated Diap1 levels in lgl mutant clones by knocking down or reducing Arf79F activity (Fig 7), is most likely due to inhibition of Yki activity and therefore elevated Hippo pathway signalling.

Minor comments:
- The authors present a well-structured manuscript, that generally is easy to understand. However, at some points, the statements given by the authors seem highly speculative.
- The figures presented in this manuscript and the statistical analysis seem adequate and are clearly described.

Response: We thank the reviewer for their support of our study. We have added more explanation to support our conclusions.

Reviewer #1 (Significance (Required)):

The study presented by Portela et al. gives new insights into the regulation of the Hippo pathway with the discovery of new proteins involved in this mechanism, which can be interesting to those working on basic research and focused on studying signal transduction. However, this study lacks some novelty.
Throughout the manuscript, the authors only observed the physiological consequences of manipulating this pathway based on the eye phenotypes, and in the discussion, many hypotheses were raised based on the already available literature, which shows that much is already known about the Hippo pathway.
The advances shown in this study are limited to the description of the signaling pathway itself and to the eye morphology. As a suggestion, the authors should explore the knowledge of their findings in order to understand how we can use them to achieve advances in other fields and physiological conditions. For example, only at the end of the discussion, did the authors raise the questions that would really push their discoveries a step forward, namely how this mechanism acts during the response to tissue wounding and whether the mammalian orthologs of Lgl and Vap33 also act via these mechanisms to control tissue growth in mammals. It would be interesting if the authors could direct their research efforts to understand if the proteins identified can be targeted to improve wound healing or to delay aging for example.
Altogether, the authors present an interesting study but, at this moment, it still lacks the significance and novelty needed for publication. We encourage the authors to keep up their good

© 2024. Published by The Company of Biologists under the terms of the Creative Commons Attribution License (https://creativecommons.org/licenses/by/4.0/).

work to address these suggestions, which will definitely improve the quality of their study.

Response: We respectfully disagree with the reviewer's comments regarding the significance of our study. On the contrary, our study is significant since it has discovered a mechanism linking Lgl and Vap33-RtGEF/Git/Arf79F and the V-ATPase to the regulation of the Hippo pathway, an important tissue growth regulatory and tumour suppressor pathway. The Drosophila eye epithelium is a highly validated model for exploring mechanisms that are relevant to human epithelial biology and cancer. Whilst extending our studies of the mechanism by which Lgl controls the Hippo pathway to wound healing and mammalian systems would be the next step, this is beyond the scope of this discovery paper.

Reviewer #2 (Evidence, reproducibility and clarity (Required)):

Summary
This manuscript investigates potential mechanisms through which the lgl gene might affect the Hippo signaling pathway. The authors employ a combination of physical interaction studies and clonal analysis in Drosophila eye discs to investigate potential links between lgl and other genes. Some of the results are intriguing, but the analysis is rather preliminary, and there are technical concerns with some of the results presented.

Main issues
- The authors propose effects of genes involved in vesicle trafficking and acidification in Hippo signaling, but there is no clear cellular mechanism described by which these effects could be mediated. This deserves further consideration. eg if they think there are effects on the localization of Hippo, this could be directly examined. In the Discussion, the authors suggest that "The V-ATPase might therefore act to inhibit Hippo pathway signalling by blocking the interaction of Lgl/Vap33/RtGEF/Git/Arf79F with Hpo in vesicles, thereby altering Hpo localization and inhibiting its activity." but Hippo is a cytoplasmic protein and has never been reported to be within vesicles.

Response: Whilst Hpo is a cytoplasmic protein there is evidence that it could also be associated with vesicles, since Hpo pathway components bind to several endocytic proteins by mass spectrometry analysis (Kwon et al., 2013, Science, PMID: **24114784**; Verghese and Moberg, 2020, Front. Cell Dev. Biol., PMID: 32010696). We have previously published that Hippo localization is altered in lgl mutant tissue (Grzeschik et al., 2010, Curr. Biol., PMID: **20362447**). For a better precision, we have updated the wording to state that the proteins described in our manuscript may alter Hippo localization "on endosomes" as opposed to the previous "in vesicles".

- The Yki stains in Fig. 1 are confusing. The nature of the signal throughout the wing disc looks very different in 1A vs 1B vs 1C, this needs to be explained or re-examined. Fig 1C (wts RNAi) seems to show an elevated Yki signal in some cells, and lower in others in - prior studies have reported that wts affects the nuclear vs cytoplasmic localization of Yki, but not its levels, so this needs to be clarified.
Response: There are some tissue folds in the eye disc tissues that might be confusing the reviewer, but Yki nuclear staining is lower in Vha68-2 mutant clones, and higher in wts knockdown and Vha44 over-expressing clones (arrowheads). When Yki is concentrated in the nucleus the staining appears more intense, as it does in the wts knockdown clones. Similar results on Yki staining upon Hippo pathway impairment in epithelial tissues have been obtained by other Hippo pathway researchers (eg PMID: **20362445,** PMID: 19900439, PMID: **19913529**, PMID: **26364751**).

- In Fig 1D the clones appear to have different effects in different regions of the eye disc; the authors should clarify. Also, the disc in 1D appears much younger than the discs in 1A-C, but similar age discs should be used for all comparisons.

Response: All eye discs are from wandering 3rd instar larvae, but the mounting of the samples on the slide and the confocal Z-section could account for apparent different regions of the eye disc showing stronger upregulation of Ex-LacZ and Yki staining. The data has been statistically analysed from multiple eye discs and the effects observed are significantly different to the control (as plotted in Fig 1E).

© 2024. Published by The Company of Biologists under the terms of the Creative Commons Attribution License (https://creativecommons.org/licenses/by/4.0/).

- The authors should clarify whether any the manipulations they perform are associated with Jnk activation, as this could potentially provide an alternative explanation for downregulation of Hippo signaling.

Response: Lgl mutant clones only upregulate the JNK target MMP1 in some cells at the border of the clones but show elevated Yki activity within the clones. Vha44 overexpressing clones do show upregulation of JNK signalling (Petzoldt et al., 2013, Dis Model Mech., PMID: **23335205**), but since JNK signalling is known to inhibit Yki activity in the eye epithelium (PMID: **22190496**), it is unlikely that the upregulation of Yki activity (downregulation of Hippo signalling) in Vha44 overexpressing clones is due to JNK activation.

- The authors report in Fig 2C,E that over-expression of Vap33 reduced expression of Diap1, which they interpret as evidence of increased Hippo pathway activity, but this experiment is lacking essential controls, as the apparent reduction of Diap1 could simply reflect increased cell death or a change in focal plane, and indeed the difference in the label stain makes it look like these cells are undergoing apoptosis. Thus it's important to also have a stain for a neutral protein, or at least a DNA stain. Additionally, it is important to stain for at least one additional marker of Hippo pathway activity (eg ex-lacZ or Yki localization), as there are other pathways that regulate Diap1

Response: We have previously examined the effect of Vap33 overexpressing clones on the Notch signalling pathway and do not see a reduction in Notch target gene expression relative to the control (Portela et al., 2018, Sci. Signal., PMID: **29871910, Fig 3**). Thus, although there might be some cell death in Vap33 overexpressing clones (possibly due to lower Diap1 levels), it is unlikely that cell death per se results in lower Diap1 levels. We are unable to conduct further experiments to examine other Hippo pathway activity markers since my lab is now closed.

- In Fig. 4 the authors perform PLA experiments to examine potential association between various pairs of proteins, but they don't show us key controls. They report in the text using single antibodies as negative controls, but this doesn't control for non-specific localization of antibodies. The better negative control is to do the PLA experiments in parallel on tissues lacking the protein being detected (eg from animals not expressing the GFP- or RFP-tagged proteins they are examining). Also, there is a lot of variation in the apparent signals shown in different PLA experiments in fig 4, the authors should comment on this.

Response: We have previously used the PLA assay to examine Lgl and Vap33 interactions (Portela et al., 2018, Sci. Signal., PMID: **29871910, Fig 2**) and have conducted an experiment expressing Vap33 tagged with HA via the GMR driver in the posterior region of the eye disc and then detected Lgl-HA protein interactions, which only showed PLA foci in the posterior region where Vap33-HA is expressed but not in the anterior region where Vap33-HA is not expressed. This may be thought of as the best possible control since these differentially expressing regions were part of the same tissue sample. Furthermore, in our previous study (Portela et al., 2018, Sci. Signal., PMID: **29871910, Fig S2**), we conducted a negative control PLA using the GFP and Vap33 antibodies in eye tissue not expressing GFP-Lgl and observed no PLA foci. We have edited the text to refer to these controls.
The variation in PLA signal may be due to low levels of expression of certain proteins or lower levels of protein-protein interactions. We have edited the text to add this explanation.

- The authors claim that RtGEF mutant cells increase Diap1 expression, and that Vap33 over-expression reverses this effect (Fig. 5). The effect of RtGEF looks very subtle and variable, it should be confirmed by examining additional reporters of Hippo pathway activity. It also seems like the disc in 5A is at a different stage &/or the quantitation is done from a different region as compared to the disc in 5C.
Response: RtGEF mutant cells have also been shown to upregulate the Yki target, Ex-LacZ (Dent et al., 2015). Unfortunately, we were unable to construct an Ex-LacZ RtGEF mutant stock and there was no available Ex antibody.
For Diap1 quantification, clones were chosen just posterior to the morphogenetic furrow of each eye disc and multiple clones were analysed relative to the adjacent wild-type clones in many samples and quantified and plotted in Fig 5E.

© 2024. Published by The Company of Biologists under the terms of the Creative Commons Attribution License (https://creativecommons.org/licenses/by/4.0/).

- The analysis of the influence of Vha68-2 mutant clones, and their genetic interaction with Git, similarly suffers from missing controls and incomplete analysis. Additional Hippo reporters besides just Diap1 should be examined. The Diap1 analysis which shows reduced expression needs examination of neutral controls or nuclear markers to assess potential apoptosis within clones, or changes in focal plane.

Response: We have also examined the effect of Vha68-2 clones on Ex-LacZ expression (Figure 1) and show that it is also reduced relative to the surrounding wild-type clones.
We have previously examined Vha68-2 mutant clones for the expression of a Notch pathway target (Portela et al., 2018, Sci. Signal., PMID: **29871910, Fig S1**) and show with DAP1 staining that cells are in the same plane and are retained in pupal retina, so are not dying. We now refer to our previous study in the text.

Similarly, the analysis of Arf79F mutant clones in Fig 7E,G is compromised by lack of controls for viability and tissue layer, and analysis of an additional Hippo reporter is once again essential.

Response: We don't believe DAPI stains are necessary as the GFP membrane/cytoplasmic staining clearly shows the outline of the cells and where the nucleus is in the mutant clones and shows that the cells are intact and not dying.

Reviewer #2 (Significance (Required)):

The strength of the study is the potential dissection of novel connections between the lgl tumor suppressor and the Hippo pathway. However, there are signifiant limitations due to the preliminary nature of the study, which is incomplete and missing essential controls.
If these limitations are overcome the work will be of interest to specialists in the field.

Response: We are hoping that our explanations and responses to the main issues above alleviate concerns regarding controls.

Reviewer #3 (Evidence, reproducibility and clarity (Required)):

In this study, Portela and colleagues identified new regulators of Hippo pathway downstream of the core apico-basal polarity protein Lgl. While the impact of Lgl depletion of Yki activation was already characterised both in Drosophila and Vertebrates, the mechanism connecting these two pathways was still unclear. Using the Drosophila eye, mosaic analysis, epistatic analysis and mass-spectrometry, they identified two routes through which Lgl depletion can lead to Hippo pathway downregulation and eye overgrowth. This regulation required the previously characterised Lgl interactor Vap33, which on the one hand activates Hippo by inhibiting the V- ATPase, and on the other hand activates Hippo through its interactions with the actin regulators Git, RtGEF (two previously characterised regulators of Hippo, https://pubmed.ncbi.nlm.nih.gov/25484297/) . They also identified another regulator of Hippo downstream of Lgl, Arf79F, whose ortholog interact with Git in mammals and is also in close proximity with Hippo, Git and RtGEF in Drosophila, and whose depletion abolish Hippo downregulation and eye overgrowth in Lgl mutant. This is a well performed study which identified new links between Lgl and regulation of the Hippo pathway. Many of them are conserved in mammals and may be relevant in pathological conditions associated with Lgl loss of function and Yap missregulation. The experiments are well conducted with a quite thorough epistatic analysis combined with many assays to characterize protein interactions. Admittedly, the molecular mechanism remains uncharacterised and some experiments may help to indicate putative mechanisms, but the characterisation of these news regulators and clear genetic interactions results constitute already solid and interesting data. I have some suggestions though that could help to reinforce the conclusions.

Main suggestions:

1. While the precise molecular mechanisms is not absolutely necessary, it would be interesting to document the subcellular localisation of these new Hippo regulators in WT and Lgl mutant context (Git, RtGEF Vap33 and Arf79F), either with Antibody if they exist, or with fusion protein

© 2024. Published by The Company of Biologists under the terms of the Creative Commons Attribution License (https://creativecommons.org/licenses/by/4.0/).

(which for a good part were already generated for the PLA results). This may reveal obvious misslocalisation which would support the role of Lgl as a scaffolding protein that maintain proper subcellular localisation of these factors.

Response: Whilst this experiment would extend the study, we are unable to do this since my lab has now closed.

2. Most of the epistatic experiments focus on factors that rescue the overgrowth and increase of diap1 expression in Lgl mutant. Did the author test if any of these core regulators are sufficient to recapitulate Lgl mutant eye phenotype, for instance Vap33 KD in the eye, or Arf79F overexpression. Negative results would still be informative as they would point to the existence of other downstream regulators of the eye phenotype

Response: Vap33 knockdown by RNAi in clones does not phenocopy the lgl mutant mosaic adult eye phenotype (Portela et al., 2018, Sci. Signal., PMID: **29871910, Fig 2**), presumably due to other functions of Vap33. We have added further details regarding this point In the Discussion. We have not examined Arf79F overexpressing clones.

3. It is at the moment hard to interpret the relevance of the results obtained by PLA. While there are some negative controls based on the absence of secondary antibody, what is the number of particle obtained for two non-interacting cortical proteins ? Since this is based on proximity, I would expect that some positive particles would still appear by chance, but much less than for two physically interacting proteins or subunits of a complex. Could the author provide such a negative control by testing for instance Git/RtGEF with another non-interacting cortical protein ? That would help to assess the relevance of the conclusions based on PLA.

Response: The PLA is a robust assay to assess protein-protein interactions of proteins that are <40nM apart and is a widely used method (PMID: **17072308;** PMID: **23921974**) and has been used previously to examine cortical protein complexes, such as the Integrin complex (PMID: **33215378**) and signalling networks (PMID: **25241761**). Whilst there might be some chance interaction of 2 cortical proteins not in a complex, proteins known not to interact have been proven not show a robust PLA signal (PMID: **25241761**). In our experiments in this study and our previous paper (Portela et al., 2018, Sci. Signal., PMID: **29871910**) we have observed robust PLA foci formation relative to negative controls in the cases where the proteins are known to interact by AP-MS and co-immunoprecipitation analysis, and no or very few foci were observed in areas of the sample where the proteins were not co-expressed. We have now added further explanation about controls to the result section.

4. Some of the epistatic links are a bit hard to interpret at the moment, and additional epistatic test may be relevant. For instance, the increase of diap1 upon Git depletion in the Vha68 mutant (Figure 6) is used to conclude that Git is required for the Hippo upregulation upon reduced V-ATPase activity. However this could be compatible with two independent branches regulating Hippo (in an opposite manner), which is more less what is suggested by the authors in their conclusion and the model of figure 8. I would suggest to reformulate this conclusion in the result part. Similarly, there is currently no experimental exploration of the epistatic link between Arf79F, Git and RtGEF (which is based on results in mammals). It would be interesting to check if Git and RtGEF mutant phenotype (Hippo downregulation) can also be suppressed by downregulation of Arf79F.

Response: We have now added further explanation to the result section regarding Fig 6. Unfortunately, we are unable to do further experiments since my lab is now closed.

5. Apart from very obvious phenotype (eye in Lgl mutant mosaic) it is a bit hard to interpret the picture of adult eye provided in this study (specially for mild phenotype). Could the authors provide more explanation in the legends, and if possible some quantitative evaluation of the phenotype when relevant? Otherwise, apart from obvious rescue of the Lgl mutant, it is a bit hard to interpret the other genotypes (e.g. : Vap33OE, RtGEF mutant, Vha68 mutant)

Response: We have added more explanation of the adult eye phenotypes in the text/fig legends.

Other minor points :

© 2024. Published by The Company of Biologists under the terms of the Creative Commons Attribution License (https://creativecommons.org/licenses/by/4.0/).

1. I would recommend when possible to clearly indicate in Figure 8 which part of the pathway are clearly documented in this study, and which part are still hypothetical (eg: link with PAK).

Response: We have re-drawn the model figure to highlight what we have found in this study by adding orange arrows between Lgl-Vap33-RtGEF/Git-Arf79F-Hpo and Lgl-Vap33-V-ATPase and V-ATPase-Hpo.

2. Page 4, the sentence "as aPKC's association with the Hpo orthologs, MST1/2, and uncoupling MST from the downstream kinase, LATS (Wts), thereby leading to increased nuclear YAP (Yki) activity [17], consistent with what we observe in Drosophila [5]." may need to be reformulated (at least I had trouble to understand it).

Response: We have edited the sentence to "In mammalian systems, deregulation of Lgl/aPKC impairs Hippo signalling and induces cell transformation, which mechanistically involves the association of aPKC with the Hpo orthologs, MST1/2, thereby uncoupling MST from the downstream kinase, LATS (Wts) and leading to increased nuclear YAP (Yki) activity [17], consistent with what we observe in Drosophila [5]."

3. Page 11 : "a decrease in Diap1 expression was observed and clones were smaller than wild-type clones (Fig 7E), suggesting that the Arf79F knockdown clones were being out-competed"
I am not sure one can conclude from this that the clone are "outcompeted" (which would suggest at context dependent disappearance of clone, while here the data could be totally compatible with a cell-autonomous decrease of growth and survival). This statement would only make sense if global eye depletion of Ar79F had no adult eye phenotype.

Response: We have edited the sentence to "a decrease in Diap1 expression was observed and clones were smaller than wild-type clones (Fig 7E), suggesting that the Arf79F knockdown clones have reduced tissue growth ----."

Reviewer #3 (Significance (Required)):

This study identifies regulators of Hippo which through their interactions with Vap33 explains for the first time how Lgl depletion leads to Hippo misregulation (without impairing apico-basal polarity). This is an interesting study based on epistatic analysis and mass-spectrometry and identify several regulators conserved in mammals. While the molecular mechanism remained to be explored, it clarifies for the first time how Lgl depletion (a core regulator of apico-basal polarity) leads to Hippo downregulation and tissue overgrowth, a phenotype also observed in mammals and characterised several years ago in Drosophila. The authors previously characterised the interaction between Vap33 and Lgl and its role in the regulation of Notch signaling through the V-ATPase. This study nicely complement these previous results and connect now Vap33 with Hippo and Lgl while answering a long unresolved question (how Lgl depletion affect Hippo pathway).

This results will be interesting for the large community studying the hippo pathway, apico-basal polarity and tissue growth. It also outlines interesting factors that could be relevant for tumour neoplasia and hyperplasia.

I have expertise in epithelial biology, apoptosis, cell competition, Drosophila, cell extrusion, mechanobiology, morphogenesis and growth regulation.

Response: We thank the reviewer for recognizing the significance of our study.

Original submission

First decision letter

MS ID#: JOCES/2023/261917

© 2024. Published by The Company of Biologists under the terms of the Creative Commons Attribution License (https://creativecommons.org/licenses/by/4.0/).

MS TITLE: The Drosophila Tumour Suppressor Lgl and Vap33 activate the Hippo pathway by a dual mechanism, involving RtGEF/Git/Arf79F and inhibition of the V-ATPase.

AUTHORS: Marta Portela, Swastik Mukherjee, Sayantanee Paul, John E La Marca, Linda M Parsons, Alexey Veraksa, and Helena E Richardson
ARTICLE TYPE: Research Article

Thank you for sending your manuscript to Journal of Cell Science through Review Commons.

It has taken longer than I would like to come to a decision because of the holiday period. It would have been nice if you could have used transcription reporters as suggested by the reviewers as I think this would have strengthened the paper's message. However, I appreciate that you cannot do any more experiments because your lab has closed. Given the situation and your responses, I am happy to tell you that your manuscript has been accepted for publication in Journal of Cell Science, pending standard ethics checks.

If you wish to see the details we hold about your manuscript or would like to view any of the files that you have submitted, decision letters or reviewers' reports on the manuscript, please click on the 'Author history' link in our Manuscript Tracking System.

© 2024. Published by The Company of Biologists under the terms of the Creative Commons Attribution License (https://creativecommons.org/licenses/by/4.0/).
